# Functional Potential of Soil Microbial Communities and Their Subcommunities Varies with Tree Mycorrhizal Type and Tree Diversity

**DOI:** 10.1128/spectrum.04578-22

**Published:** 2023-03-23

**Authors:** Bala Singavarapu, Jianqing Du, Rémy Beugnon, Simone Cesarz, Nico Eisenhauer, Kai Xue, Yanfen Wang, Helge Bruelheide, Tesfaye Wubet

**Affiliations:** a Department of Community Ecology, UFZ-Helmholtz Centre for Environmental Research, Halle, Germany; b Institute of Biology/Geobotany and Botanical Garden, Martin Luther University Halle-Wittenberg, Halle, Germany; c German Centre for Integrative Biodiversity Research (iDiv), Leipzig, Germany; d Leipzig Institute for Meteorology, Universität Leipzig, Leipzig, Germany; e CEFE, Université Montpellier, CNRS, EPHE, IRD, Montpellier, France; f Institute of Biology, Leipzig University, Leipzig, Germany; g College of Resources and Environment, University of Chinese Academy of Sciences, Beijing, China; h Beijing Yanshan Earth Critical Zone National Research Station, University of Chinese Academy of Sciences, Beijing, China; i State Key Laboratory of Tibetan Plateau Earth System, Environment and Resources, Chinese Academy of Sciences, Beijing, China; State Key Laboratory of Mycology, Institute of Microbiology, Chinese Academy of Sciences

**Keywords:** co-occurrence network, microbial subcommunities, nutrient cycling, functional potential, tree mycorrhizal type, tree diversity

## Abstract

Soil microbial communities play crucial roles in the earth’s biogeochemical cycles. Yet, their genomic potential for nutrient cycling in association with tree mycorrhizal type and tree-tree interactions remained unclear, especially in diverse tree communities. Here, we studied the genomic potential of soil fungi and bacteria with arbuscular (AM) and ectomycorrhizal (EcM) conspecific tree species pairs (TSPs) at three tree diversity levels in a subtropical tree diversity experiment (BEF-China). The soil fungi and bacteria of the TSPs’ interaction zone were characterized by amplicon sequencing, and their subcommunities were determined using a microbial interkingdom co-occurrence network approach. Their potential genomic functions were predicted with regard to the three major nutrients carbon (C), nitrogen (N), and phosphorus (P) and their combinations. We found the microbial subcommunities that were significantly responding to different soil characteristics. The tree mycorrhizal type significantly influenced the functional composition of these co-occurring subcommunities in monospecific stands and two-tree-species mixtures but not in mixtures with more than three tree species (here multi-tree-species mixtures). Differentiation of subcommunities was driven by differentially abundant taxa producing different sets of nutrient cycling enzymes across the tree diversity levels, predominantly enzymes of the P (*n* = 11 and 16) cycles, followed by the N (*n* = 9) and C (*n* = 9) cycles, in monospecific stands and two-tree-species mixtures, respectively. Fungi of the Agaricomycetes, Sordariomycetes, Eurotiomycetes, and Leotiomycetes and bacteria of the *Verrucomicrobia*, *Acidobacteria*, *Alphaproteobacteria*, and *Actinobacteria* were the major differential contributors (48% to 62%) to the nutrient cycling functional abundances of soil microbial communities across tree diversity levels. Our study demonstrated the versatility and significance of microbial subcommunities in different soil nutrient cycling processes of forest ecosystems.

**IMPORTANCE** Loss of multifunctional microbial communities can negatively affect ecosystem services, especially forest soil nutrient cycling. Therefore, exploration of the genomic potential of soil microbial communities, particularly their constituting subcommunities and taxa for nutrient cycling, is vital to get an in-depth mechanistic understanding for better management of forest soil ecosystems. This study revealed soil microbes with rich nutrient cycling potential, organized in subcommunities that are functionally resilient and abundant. Such microbial communities mainly found in multi-tree-species mixtures associated with different mycorrhizal partners can foster soil microbiome stability. A stable and functionally rich soil microbiome is involved in the cycling of nutrients, such as carbon, nitrogen, and phosphorus, and their combinations could have positive effects on ecosystem functioning, including increased forest productivity. The new findings could be highly relevant for afforestation and reforestation regimes, notably in the face of growing deforestation and global warming scenarios.

## INTRODUCTION

Microorganisms, especially bacteria and fungi, contribute enormously to terrestrial ecosystem services: for example, by playing a vital role in soil nutrient cycling (1–4). Particularly, the contribution of plant symbiotic microbes in soil nutrient cycling has been well reported. For example, mycorrhizal fungi form symbiotic associations with around 90% of terrestrial plant species and take part in nutrient cycling by mobilizing nitrogen (N) and phosphorus (P) in soils (5, 6). Similarly, plant-symbiotic bacteria belonging to *Rhizobium* and *Frankia* can fix nitrogen and thus essentially participate in N cycling (7). Moreover, at the community level, it is also important to consider the extensive contribution of free-living soil bacteria and fungi to soil nutrient cycling as they constitute a major part of soil microbiota (8). A few examples include carbon-fixing *Actinobacteria* (9, 10), nitrogen-fixing *Azotobacter* (9, 10), and phosphate-solubilizing *Acidobacteria* (11, 12). Likewise, *Penicillium*, Aspergillus, and *Trichoderma* are free-living fungi and known for being actively involved in the decomposition of soil organic compounds (C cycle), nitrification (N cycle), and P solubilization (P cycle), respectively (13–15).

Soil stoichiometry of nutrients like C/N/P ratios is known to affect the soil microbial communities, depending upon their constituting members’ organismal nutrient stoichiometric ratios (16, 17). For example, it was reported that high N and P abundances in soil favor the abundance of fast-growing bacteria (i.e., copiotrophic, *r*-strategists) like *Actinobacteria* and *Alphaproteobacteria* while discriminating against slow-growing bacteria (i.e., oligotrophic, *K*-strategists) like *Acidobacteria* (18, 19). Also, previous research suggests that ectomycorrhizal fungi (EMF) preferentially associate with soils of high-C/N substrates, whereas saprotrophic fungi prevail in soils with low C/N ratios (20–22). There has been a surge in recent studies showing the link between microbial diversity, community composition, and soil ecosystem multifunctionality (23–27). However, there is still a knowledge gap about how the soil microbial communities vary in the stoichiometry of their nutrient cycling genomic potential, which can be the relative combinations of genes coding for different nutrient cycling enzymes. In a study taking a genomic perspective on soil carbon cycling, Hartman et al. (28) reported links between microbial community composition, the microbe’s C, N, and P substrate utilization potential, and C turnover. This highlights the importance of studying the genomic potential of microbial communities to better understand soil nutrient cycling.

Given the fact that soil C, N, and P cycles are linked, it is essential to study the co-occurring bacterial and fungal communities together for their genomic potential in the cycling of different major nutrients and their combinations (*viz.* C, N, P, CN, CP, NP, and CNP). For instance, the ability to decompose soil organic matter (SOM) with various nutrient ratios depends on the composition of soil microbial communities (29). Subsequently, the decomposed SOM would be available for bacteria and fungi conditioned on their abilities to continue with either N fixation or denitrification (30, 31) and/or concurrently also be available for P mineralization or solubilization (32, 33). This linkage between different soil nutrient cycling processes and the different microbes involved can be viewed from a “microbial syntrophy” (microbial metabolic interrelationships) perspective (34), which is affected by many factors (for example, available nutrient ratios, etc.) but essentially depends on the genomic potential of the members of the microbial communities.

The ecological processes and relationships within a microbial community can cumulatively emerge from the constituting microbial groups/clusters (i.e., taxa that are more strongly associated within that group than with other groups), which are also known as subcommunities (35, 36). Based on network theory, the study of subcommunities, also known as modules, can provide key insights into the overall functioning of the microbial community, allowing us to assess the metabolic potential based on the single microbes’ functional roles, which otherwise remains a black box. In addition, knowledge of subcommunities also sheds light on the ecological processes that shape and regulate community structure and organization, such as environmental filtering or niche differentiation (37). For example, recent studies in soil microbial ecology have taken the advantage of subcommunity-based analyses to develop a deeper understanding of environment-specific relationships (38, 39) and the functional roles of microbial communities (40–42).

One of the key factors influencing the soil microbial communities in forests is the tree mycorrhizal type (43), which is also known to impact microbial functional genes (44) and soil nutrient cycling (45). In addition, tree diversity has also been reported to affect soil microbial communities (46–48) and soil nutrient availability (49). Despite these efforts, there is still a great need to understand how the tree mycorrhizal type and tree diversity affect the co-occurring soil bacterial and fungal communities at the subcommunity level and, in consequence, their genomic functional potential for nutrient cycling. Insight into these processes would provide a broader understanding of the intrinsic characteristics of soil microbial groups operating in ecological processes and the functional potential emerging at the community level. Such in-depth mechanistic understanding would also be the basis for managing forest soil ecosystems to maintain or increase forest multifunctionality.

To fill this knowledge gap, this study was conducted at the BEF-China experimental research platform (50), using tree species of two mycorrhizal types, namely, ectomycorrhizal (EcM) and arbuscular mycorrhizal (AM), at different tree diversity levels (43). We employed the fungal-bacterial interkingdom co-occurrence network approach (51) to derive the microbial subcommunities (here, interchangeably used with “modules”) and used PICRUSt2 (52) to predict the potential genomic functions with regard to nutrient cycling from the amplicon sequencing data. Our main objective was to understand how the stoichiometry in genomic functional potential of soil microbial communities and their subcommunities with regard to the three major nutrient cycles and their combinations (C, N, P, CN, CP, NP, and CNP) varies in EcM and AM trees at different tree diversity levels. In particular, we asked the following research questions.

1. How do the EcM and AM tree species pair (TSP) soil bacterial and fungal community co-occurrence network structures differ across tree diversity levels, and which soil characteristics drive the composition of the subcommunities in these networks?
2. What are the effects of tree diversity and tree mycorrhizal type on the predicted genomic functional potential (in terms of C, N, and P cycles and their combinations) of the co-occurring bacterial and fungal communities?
3. How do EcM and AM TSPs soil microbial subcommunities differ in their genomic functional abundances in the three nutrient cycles and their combinations within the tree diversity levels, and which microbial taxa drive these differences?

## RESULTS

### EcM and AM TSPs soil microbial interkingdom network characteristics.

**FIG 1:**
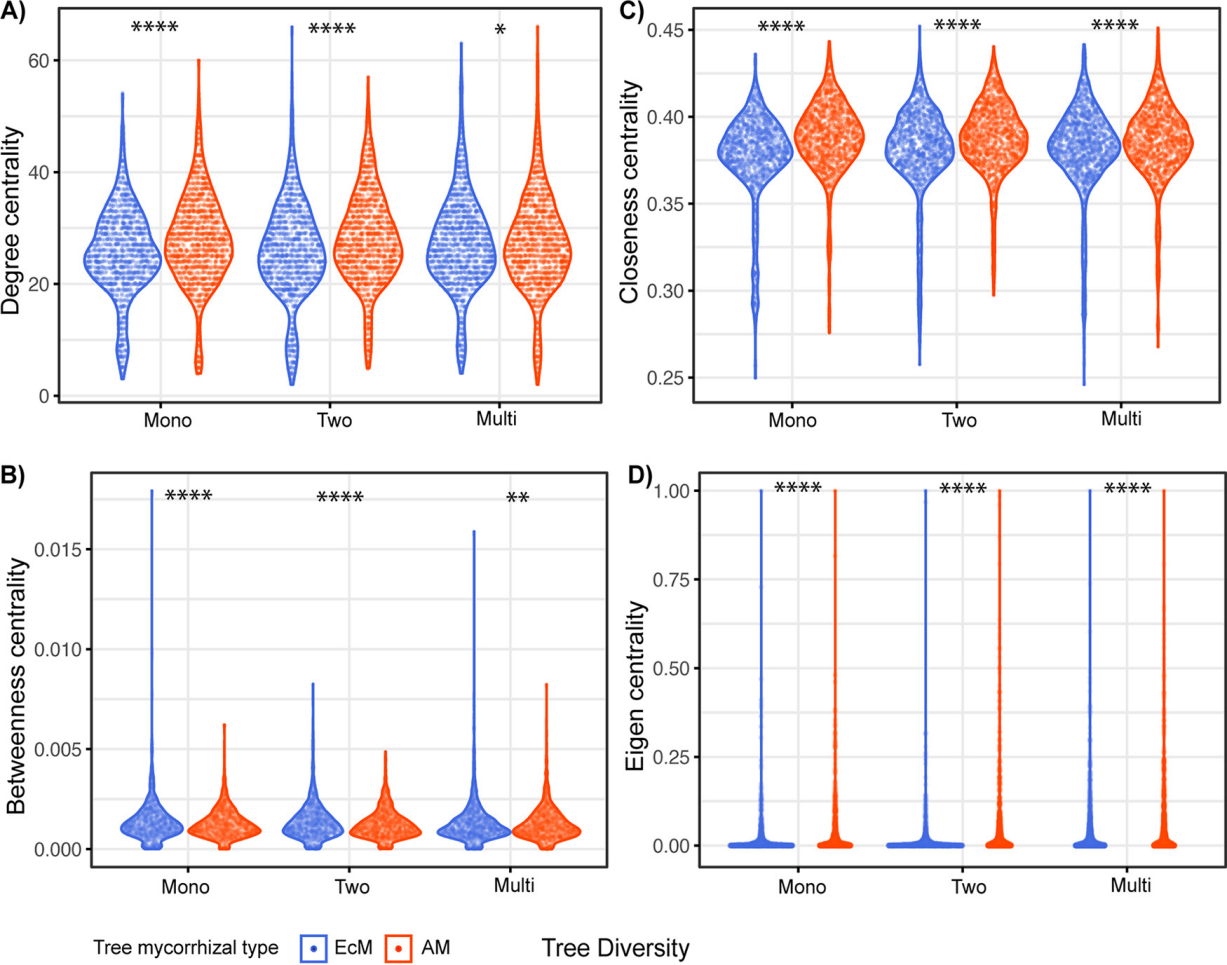
Comparison of distribution of EcM and AM interkingdom network centrality indices along the tree diversity levels. On the *y* axis are shown centrality indices, and on the *x* axis are shown the EcM and AM TSPs and the tree diversity levels (“Mono” for monospecific stands, “Two” for two-tree-species mixtures, and “Multi” for multi-tree-species mixtures). (A) Node degree centrality; (B) betweenness centrality; (C) closeness centrality; (D) eigenvector centrality. The asterisks show the *P* value significance level: *, *P* ≤ 0.05; **, *P* ≤ 0.01; ****, *P* ≤ 0.0001.

The differences in the number of input bacterial taxa used for the construction of networks at each tree diversity level were minuscule between EcM and AM trees (ranging from 796 to 798 amplicon sequence variants [ASVs]). The fungal input varied most in two-tree-species mixtures, with 430 and 503 ASVs for EcM and AM networks, respectively (see Table S1 in the supplemental material). Consistently we found no contrasting differences in clustering coefficient and modularity; however, there are three more modules in the EcM than AM network in each of the monospecific stands and two-tree-species tree diversity levels (Table S1). To assess the underlying network community organization and also the importance of the community members, we tested the distribution of four important network centrality indices, namely, node degree (used to identify community hub taxa), betweenness (a measure of a taxon’s influence in the network), closeness (a measure of the closeness of a taxon to all other members), and eigenvector centrality (a measure of a taxon’s linkage to others accounting for how connected the others are). We found significant differences (*P* < 0.05) in the distributions of these four centrality indices between EcM and AM networks in all tree diversity levels (Fig. 1). AM networks had higher median values of these distributions except for betweenness centrality, wherein EcM networks had higher values, especially at the monospecific stands and two-tree-species tree diversity levels, indicating differences in the organization of microbial taxa in their respective communities (Fig. 1).

### Subcommunities significantly responding to the soil environment.

We identified the subcommunities of all EcM and AM networks that were significantly associated with the soil variables using the distance-based redundancy analysis (dbRDA) models (Table S2). Overall, 21 of the 43 identified modules were found to be significantly responsive to the soil environment. For AM, 4 (out of 5), 4 (out of 6), and 3 (out of 8) significant modules were found in the monospecific stands, two-tree-species, and multi-tree-species (i.e., ≥4 tree species) mixtures, respectively, and for EcM, 3 (out of 8), 4 (out of 9), and 3 (out of 7) significant modules were found, respectively. Except for one AM module in two-tree-species mixtures, all of the significant modules (both AM and EcM) were strongly pH sensitive. We found one AM module in each of the tree diversity levels associated with nitrate, while in EcM communities, all modules in two-tree-species mixtures were associated with nitrate in addition to a module in monospecific stands. Although all of the significant AM modules in monospecific stands were related to P, this was only the case for one of the EcM modules (*F *= 2.09; *P* = 0.04). Furthermore, one module of each EcM (*F *= 1.51, *P* = 0.04) and AM (*F *= 1.56, *P* = 0.03) network in monospecific stands was associated with C. Total N and NH_4_^+^ were found to be significantly related to both EcM and AM modules in two-tree-species mixtures. In multi-tree-species mixtures, AM modules were significantly related to NO_3_^−^ and moisture in addition to pH, which was the only significant soil variable associated with EcM modules. Collectively, this indicated the differential roles of different subcommunities of AM and EcM networks in different tree diversity levels.

### Tree mycorrhizal type and tree diversity-level effects on the predicted functional potential of co-occurring bacterial and fungal communities.

**FIG 2:**
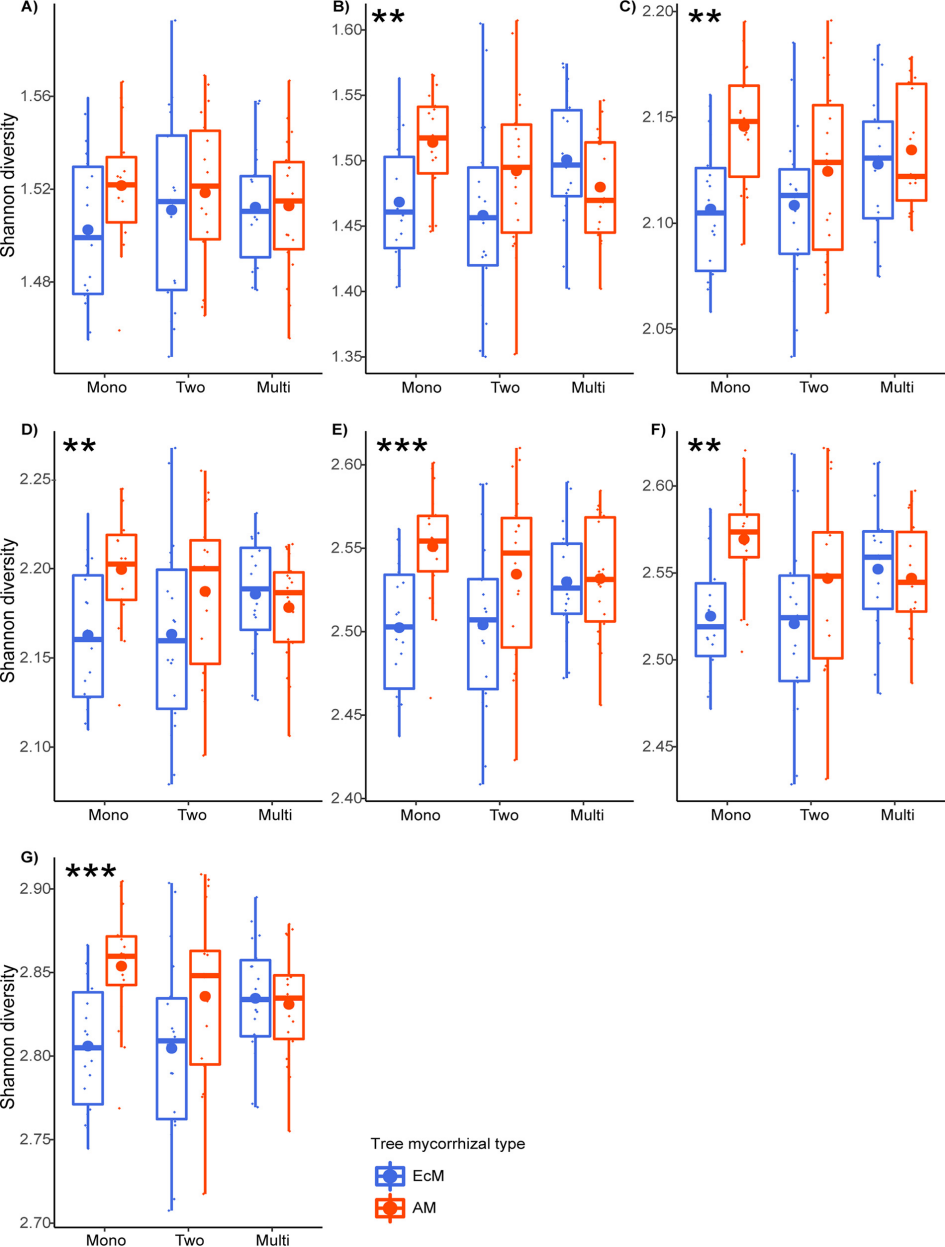
Comparison of functional diversity of EcM and AM TSP soil microbial communities along the tree diversity levels. On the *y* axis is shown the Shannon diversity index, and on the *x* axis are shown the EcM and AM TSPs and the tree diversity levels (“Mono” for monospecific stands, “Two” for two-tree-species mixtures, and “Multi” for multi-tree-species mixtures). (A) Carbon; (B) nitrogen; (C) phosphorus; (D) carbon and nitrogen; (E) carbon and phosphorus (F); nitrogen and phosphorus; (G) carbon, nitrogen, and phosphorus. The asterisks show the *P* value significance level: **, *P* ≤ 0.01; ***, *P* ≤ 0.001.

In total, 57 nutrient cycling-related EC numbers known to be part of the C, N, and P cycles were used to filter the PICRUSt2 predicted gene family content for both bacterial and fungal data sets that were used to construct the co-occurrence networks (Table S3). We found a total of 64 (43 for bacteria and 21 for fungi) ECs, where the functional abundance matrix contained 45 unique ECs comprised of 11, 16, and 18 enzymes related to C, N, and P cycling, respectively (Table S4). Significant effects of the tree mycorrhizal type were observed on the functional diversity of the co-occurring microbial community in all nutrient cycling combinations, except for C, N, and CN. In contrast, the effects of tree diversity and the interaction with mycorrhizal type were not significant in any of the nutrient cycling combinations (Table S5). Moreover, the *post hoc* analysis revealed that a tree mycorrhizal type effect was only present in monospecific stands (except for C), but was absent in two-tree-species and multi-tree-species mixtures (Fig. 2).

Permutational multivariate analysis of variance (PERMANOVA) of the effects of tree mycorrhizal type and tree diversity level on the microbial community genomic functional potential of nutrient cycling combinations showed a strong effect of tree mycorrhizal type on all combinations of genomic functional compositions (*R*^2^ value range, 5.5 to 12.8%). In addition, significant interaction effects of tree mycorrhizal type and tree diversity were found for CP and CNP combinations (Table 1). Furthermore, *post hoc* analysis of the whole community revealed that the tree mycorrhizal type effect was not significant in multi-tree-species mixtures (Table S6). Comparative analysis of the functional compositions of the whole community with those of the significantly soil-responsive modules showed similar results, except for the additional significance of interaction terms for CN and NP (Table S7). Similarly, the tree mycorrhizal type effect was also not significant in multi-tree-species mixtures (Table S8).

**TABLE 1:** Effects of tree mycorrhizal type and tree diversity level on the nutrient cycling functional compositional differences of co-occurring soil fungal and bacterial communities based on PERMANOVA with 999 permutations

Nutrient cycle	Factor	df	*F*	*R* ^2^	*P*_adj_ ^*a*^
C	Mycorrhizal_Type (M)	1	6.281	0.055	0.003**
	Tree_Diversity (L)	2	1.097	0.019	0.488
	Interaction (M × L)	2	1.553	0.027	0.209

N	Mycorrhizal_Type (M)	1	15.663	0.128	0.003**
	Tree_Diversity (L)	2	0.504	0.008	0.707
	Interaction (M × L)	2	2.067	0.034	0.192

P	Mycorrhizal_Type (M)	1	14.342	0.116	0.003**
	Tree_Diversity (L)	2	1.05	0.017	0.488
	Interaction (M × L)	2	2.438	0.04	0.092

CN	Mycorrhizal_Type (M)	1	11.902	0.1	0.003**
	Tree_Diversity (L)	2	0.617	0.01	0.707
	Interaction (M × L)	2	2.184	0.037	0.103

CP	Mycorrhizal_Type (M)	1	14.789	0.117	0.003**
	Tree_Diversity (L)	2	0.619	0.01	0.707
	Interaction (M × L)	2	3.938	0.063	0.021*

NP	Mycorrhizal_Type (M)	1	15.158	0.122	0.003**
	Tree_Diversity (L)	2	0.615	0.01	0.707
	Interaction (M × L)	2	2.679	0.043	0.092

CNP	Mycorrhizal_Type (M)	1	15.022	0.12	0.003**
	Tree_Diversity (L)	2	0.544	0.009	0.707
	Interaction (M × L)	2	3.353	0.054	0.042*

a All significant adjusted *P* values (*P*_adj_) are highlighted in boldface followed by the significance level: *, *P* ≤ 0.05; **, *P* ≤ 0.01.

### Pairwise comparison of functional abundances of EcM and AM TSPs’ soil microbial subcommunities.

**FIG 3:**
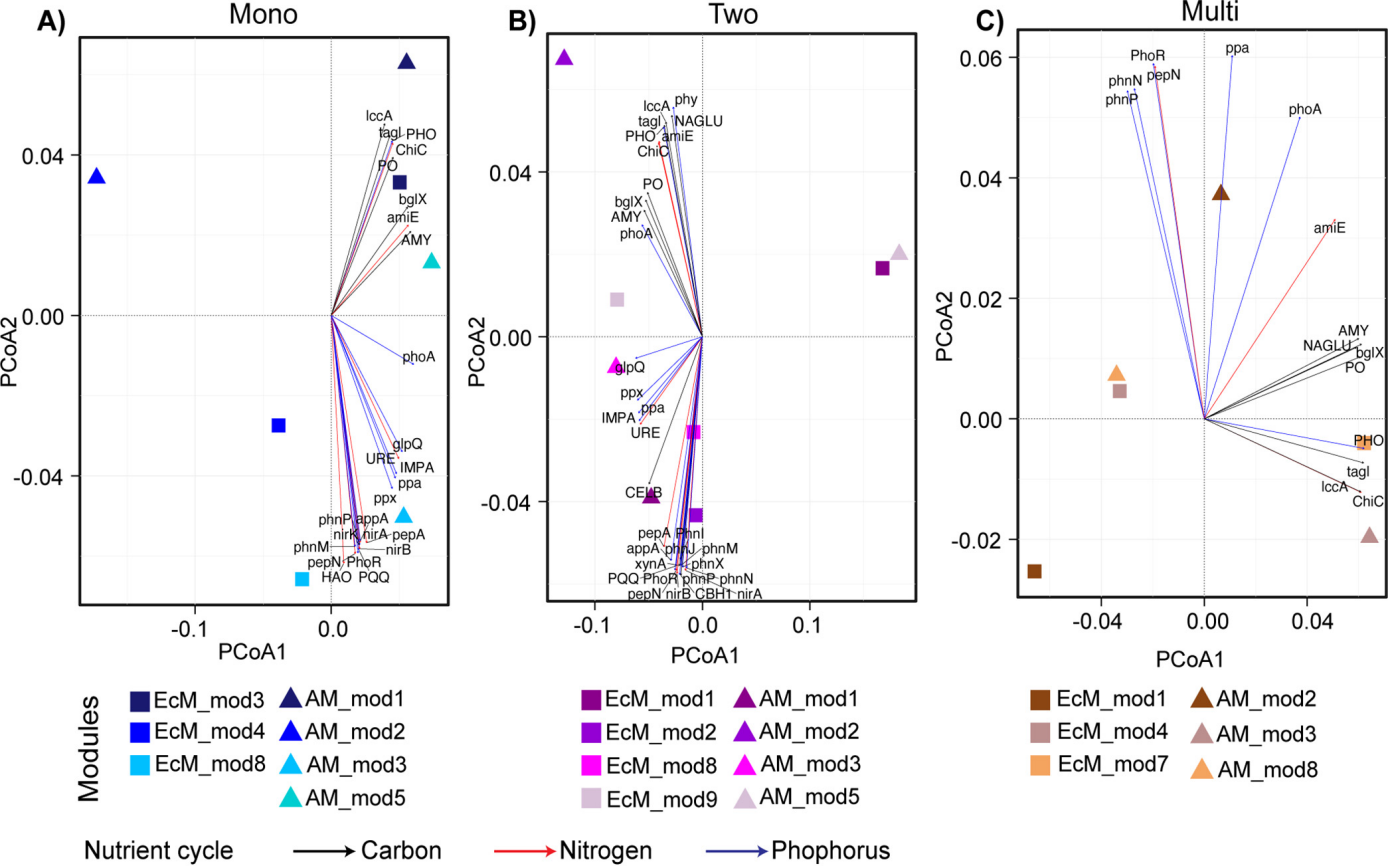
Principal-coordinate analysis (PCoA) of EcM and AM modules along the tree diversity levels. (A) Monospecific stands; (B) Two-tree species mixtures; (C) Multi-tree species mixtures. The full names for the abbreviations of enzymes are as follows: PQQ, quinoprotein glucose dehydrogenase; nirB, nitrite reductase (NADH); nirK, nitrite reductase (NO-forming); nirA, ferredoxin-nitrite reductase; HAO, hydroxylamine reductase; PhoR, histidine kinase; tagl, triacylglycerol lipase; PhoA, alkaline phosphatase; PHO, acid phosphatase; IMPA, inositol-phosphate phosphatase; appA, 4-phytase; glpQ, glycerophosphodiester phosphodiesterase; phnP, phosphoribosyl 1,2-cyclic phosphate phosphodiesterase; AMY, α-amylase; ChiC, chitinase; bglX, β-glucosidase; pepA, leucyl aminopeptidase; pepN, membrane alanyl aminopeptidase; amiE, amidase; URE, urease; ppa, inorganic diphosphatase; ppx, exopolyphosphatase; phnM, α-d-ribose 1-methylphosphonate 5-triphosphate diphosphatase; lccA, laccase; PO, peroxidase; phnN, ribose 1,5-bisphosphate phosphokinase; phnI, α-d-ribose 1-methylphosphonate 5-triphosphate synthase; phym 3-phytase; phnX, phosphonoacetaldehyde hydrolase; CELB, cellulase; NAGLU, α-*N*-acetylglucosaminidase; xynA, endo-1,4-β-xylanase; CBH1, cellulose 1,4-β-cellobiosidase; phnJ, α-d-ribose 1-methylphosphonate 5-phosphate C-P-lyase.

The principal coordinate analysis (PCoA) ordination based on the relative functional abundances showed that the significant subcommunities of EcM and AM TSPs soil microbial networks became decreasingly distant from monospecific stands to two-tree-species and multi-tree-species mixtures (Fig. 3). In addition, envfit analysis (*P* < 0.01) indicated that the differentiation of these subcommunities might be driven by the different sets of nutrient cycling enzymes across the tree diversity levels, predominantly by enzymes of the P cycle (Table S9). In monospecific stands, the significantly correlated enzymes were predominantly related to P (*n* = 11), followed by N (*n* = 9) cycles, while in two-tree-species mixtures, they were related to P (*n* = 16), followed by C (*n* = 9) cycles. In contrast, in multi-tree-species mixtures, fewer enzymes were correlated with the differentiation of modules, and those were mainly related to the C (*n* = 6) and P (*n* = 6) cycles (Table S9).

**FIG 4:**
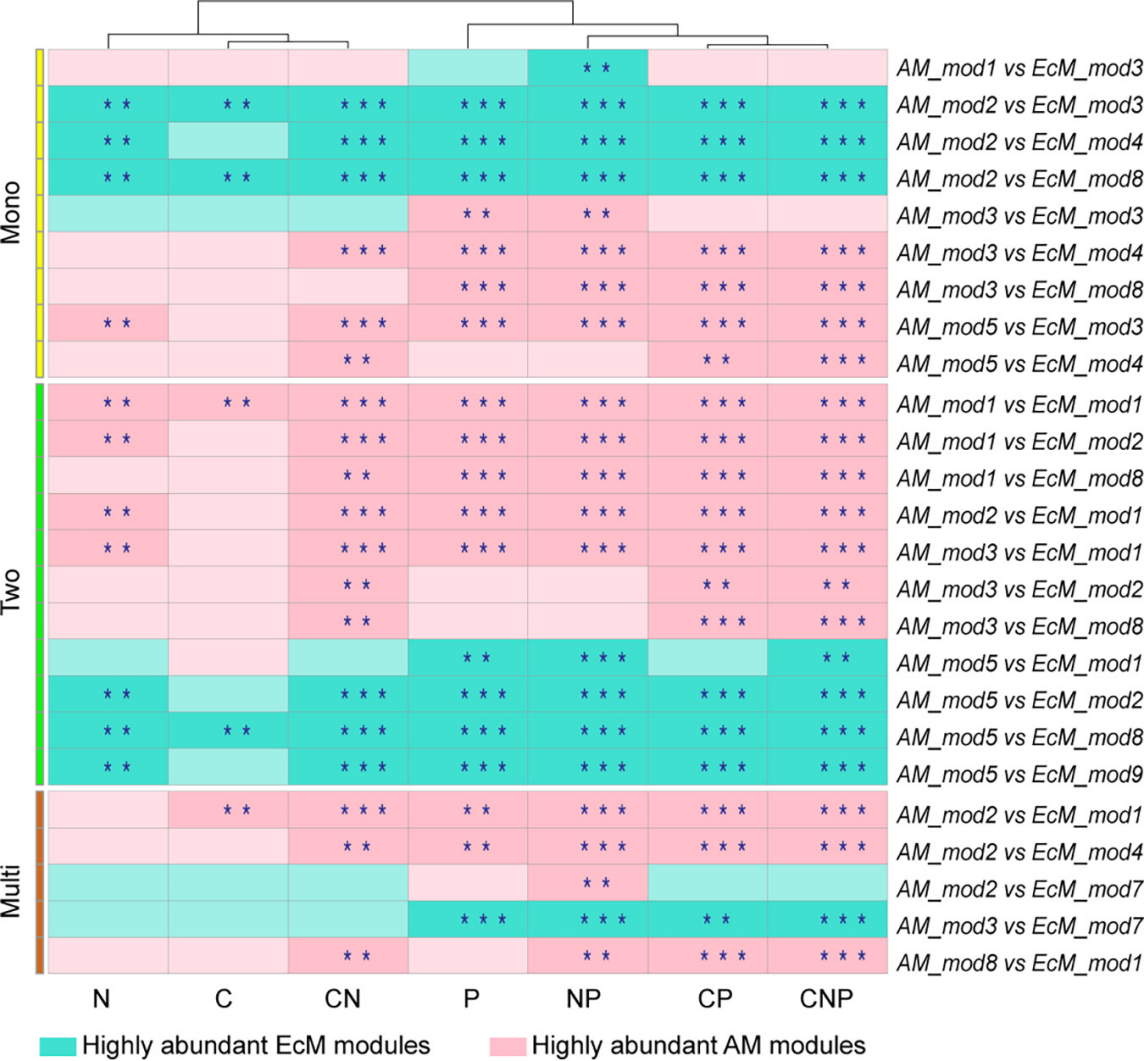
Heat map of pairwise comparisons of EcM and AM modules along the tree diversity levels (“Mono” for monospecific stands, “Two” for two-tree-species mixtures, and “Multi” for multi-tree-species mixtures). The asterisks show the *P* value significance level: **, *P* ≤ 0.01; ***, *P* ≤ 0.001.

Furthermore, pairwise comparisons across the significant subcommunities of EcM and AM TSP soil microbial networks revealed that 25 module pairs were significantly different in terms of their genomic potential for nutrient cycling. Except for C and N, in all nutrient cycling combinations, we found a higher number of significantly abundant AM modules across the tree diversity levels (Fig. 4). Interestingly, no significant differences were found in N-cycling potential in multi-tree-species mixtures. Furthermore, for C-related gene families, only EcM modules were significantly abundant in monospecific stands, while for C and CN combinations in multi-tree-species mixtures, AM modules were significantly abundant (Fig. 4). In addition, the pairwise comparisons of significant modules within tree mycorrhizal type (i.e., AM versus AM and EcM versus EcM modules) indicated that the proportion of significant differences was higher in AM subcommunities in all combinations, except for CNP (equal proportion), compared to EcM subcommunities (see Fig. S1 in the supplemental material).

### Differentially abundant taxa behind the observed functional abundance differences of EcM and AM TSPs’ soil microbial subcommunities.

**FIG 5:**
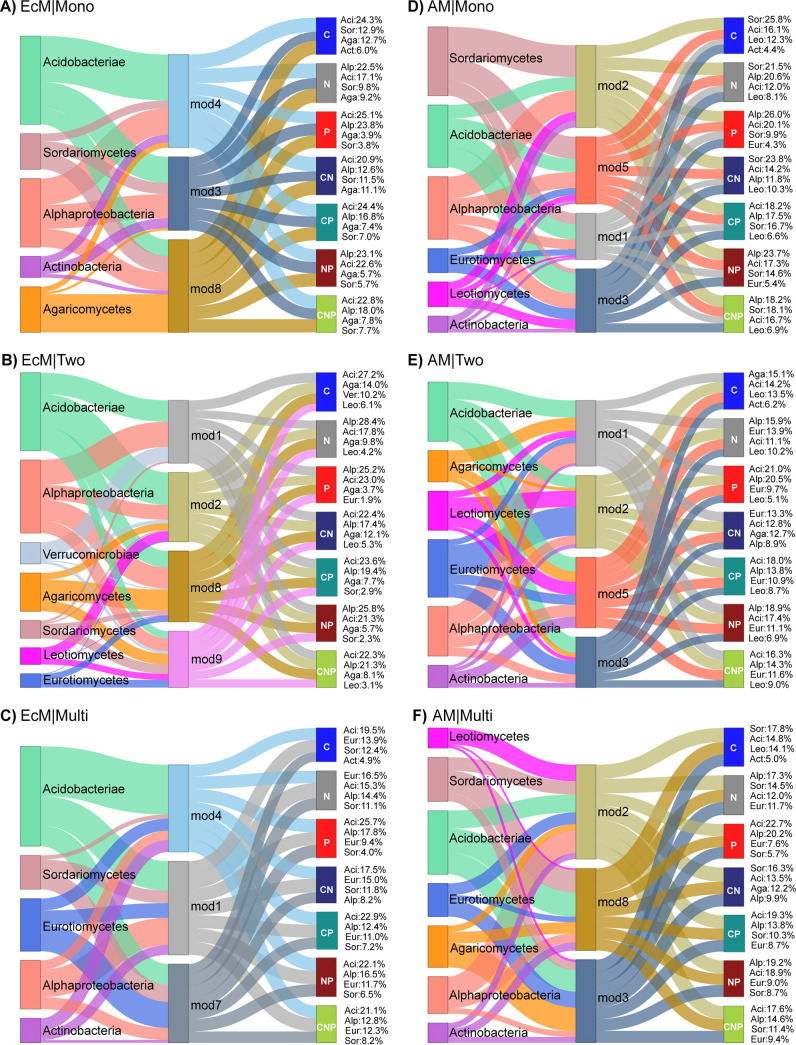
Sankey plots showing the top differentially abundant taxa from each of the EcM and AM networks along the tree diversity levels and their proportional contributions to the functional abundances. (A to C) EcM networks; (D to F) AM networks. Connections (edges) represent the proportion of relative functional abundances of each top two bacterial and fungal taxa and their distribution in each of the soil-responsive subcommunities of the EcM and AM networks. The text beside the C, N, P, CN, CP, NP, and CNP nodes denotes the top two bacterial and fungal taxa per network contributing to the respective nutrient combinations. Aci, *Acidobacteria*; Sor, Sordariomycetes; Ver, *Verrucomicrobia*; Alp, *Alphaproteobacteria*; Agr, Agaricomycetes; Eur, Eurotiomycetes; Leo, Leotiomycetes; Act, *Actinobacteria*.

We tested the differences in relative functional abundances of taxa between each EcM and AM significantly soil-responding module pairs within each tree diversity level and found a total of 995 unique differentially abundant ASVs. Furthermore, all the ASVs were aggregated at the class taxonomic level, and we identified the two most differentially abundant classes in both bacteria and fungi that strongly contributed to the functional abundances of EcM and AM TSP soil microbial communities at each tree diversity level for all nutrient cycling combinations (Fig. 5). These contributions ranged from 48% to 62% of the relative functional abundances. In monospecific stands for EcM modules, Agaricomycetes and Sordariomycetes were the predominant fungi contributing to the functional abundances of all nutrient cycling combinations. In AM modules, Sordariomycetes were the top fungi, followed by Leotiomycetes, contributing to all nutrient cycling combinations except for P (4.3%) and NP (5.4%) combinations, while Eurotiomycetes were the second most important. In the case of bacteria, *Acidobacteria* and *Alphaproteobacteria* were the predominant contributors in both EcM and AM modules, except to the C cycle, not only in monospecific stands but also in two- and multi-tree-species mixtures. Interestingly, *Actinobacteria* were the second most important contributor to the C cycle across the tree diversity levels, except in EcM modules of two-tree-species mixtures, where *Verrucomicrobia* (10.2%) took that place. In two-tree species mixtures, for EcM modules, Agaricomycetes were the predominant fungal contributor to all nutrient cycling combinations, followed by Leotiomycetes in C, N, CN, and CNP combinations, Sordariomycetes in CP (3%) and NP (2.3%), and Eurotiomycetes (1.9%) in the P cycle, while for AM modules, Eurotiomycetes, followed by Leotiomycetes, were the major contributors to most of the nutrient cycling combinations, except in C (15.1%) and CN (12.7%), where Agaricomycetes were predominant. In multi-tree-species mixtures, Eurotiomycetes followed by Sordariomycetes were the main fungal contributors to all nutrient cycling combinations in EcM modules. This was also the case for AM modules, except for the C and CN combinations, wherein Leotiomycetes and Agaricomycetes were the second major contributors, respectively. Across the tree diversity levels, in both EcM and AM modules, bacteria outweighed fungi as major differentially abundant contributors to the P cycle. Furthermore, compared to EcM, higher fungal contribution in AM modules was found in monospecific stands and two-tree-species mixtures (Fig. 5).

## DISCUSSION

### EcM and AM TSPs soil microbial interkingdom networks and their subcommunities differ in their ecological properties.

The network topological parameters provide key insights into the associations between taxa and the influence of some taxa on particular modules or the whole community. In our study, the observed significant differences between EcM and AM TSP soil microbial co-occurrence networks revealed differences in the taxon assembly and organization in the respective communities. Similarly, a recent greenhouse experimental study, Yuan et al. (53) reported significant differences in the co-occurrence network topology between arbuscular mycorrhizal fungal (AMF)-bacterial networks and nonmycorrhizal fungal (comprising saprotrophs, pathogens, endophytes, and unclassified)-bacterial networks. Relatively high values of degree centrality and betweenness centrality may indicate stronger relationships among the taxa and a powerful influence of some taxa on bridging or communicating between different parts of the network, respectively (54). Our results show that EcM TSPs’ soil microbial networks had relatively higher betweenness centrality than that of AM networks, especially in monospecific stands and two-tree-species mixtures, suggesting that some key taxa might exert control over other taxa members of the network. A relatively higher abundance of ectomycorrhizal fungi (EMF) in EcM TSPs’ soils which were known to regulate other microbes in the community (44, 55) might be a possible reason for the higher betweenness centrality. In contrast, the higher degree centrality in AM networks, especially in monospecific stands and two-tree species mixtures could be attributed to the relatively higher abundance of saprotrophs in AM TSP soils (56).

Microbes belonging to a subcommunity/module may share similar ecological processes like nutrient cycling functions or be affected by the same environmental filtering processes (37, 41). In our analysis, we identified such modules: for instance, in AM monospecific stands, all of the modules had significant relationships with P, which is compliant with the fact that AM trees acquire P through the arbuscular mycorrhizal fungi (AMF), and P is a limiting nutrient for the soil microbes in the subtropical systems with AM-dominated stands (57). Interestingly, the modules (both EcM and AM) in two-tree-species mixtures were strongly related to N or its inorganic forms, NO_3_^−^ and NH_4_^+^. It is well known that N is a vital limiting nutrient for both plants and microbes (58) and that the EcM and AM tree-dominated systems have contrasting N acquisition and allocation strategies, where organic N is preferred in EcM systems, while this is the case for inorganic N in AM systems (59). One possible reason for the observed association of modules with N or the inorganic N compounds in two-tree-species mixtures could be the coexistence of different mycorrhizal type trees in a plot (i.e., AM tree species with EcM trees and vice versa). This proportional addition of contrasting N-acquisitioning tree individuals in one plot would have triggered the mechanisms that may limit the preferred source of N for the associated soil microbial subcommunities. In multi-tree-species mixtures, all EcM and AM modules were significantly associated with pH, which is known to affect both bacterial and fungal communities (60, 61) and has a subtle relationship with soil nutrients. For example, low pH was reported to impede N mineralization and nitrification (5, 62, 63), while P availability was suggested to be high at near-neutral pH: i.e., pH 6.5 to 7 (64 [but see reference 65]). Consequently, the microbial subcommunities in multi-tree-species mixtures might have dynamic functional roles in nutrient cycling.

### Functional potentials of EcM and AM TSP soil co-occurring bacterial and fungal communities were strongly impacted by tree mycorrhizal type.

As expected, we found a significant tree mycorrhizal type effect on the functional compositions of the co-occurring microbial communities. Our results are in line with a study from boreal and temperate regional sites by Bahram et al. (44), who reported significant differences in the composition of microbial functional genes between sites dominated by EcM and AM mycorrhizal type plants. Through their specific mycorrhizal partners, trees can select the associated microbial communities with the required functional abilities (66–68). For example, given the genomic potential to release oxidative and hydrolytic extracellular enzymes to directly break down the soil organic matter (6, 59), EMF have been reported to outcompete and limit the saprotrophs in microbial communities of EcM tree-dominated systems (69). In contrast, AMF are known to have very little genomic repertoire for enzymatic degradation of soil organic matter. In consequence, they rely upon and enrich saprotrophic fungi and bacteria in soils under AM trees (22, 70). Furthermore, we found significant interactive effects of tree diversity and tree mycorrhizal type in some nutrient cycling combinations (CP and CNP for whole communities and CN, NP, CP, CNP for significant modules), wherein multi-tree-species mixtures neutralize the tree mycorrhizal type effect on the functional compositions of soil microbial communities. More co-occurring tree species and including different mycorrhizal type trees in multi-tree-species mixtures could be the potential explanation for the observed absence of significant differences in the functional compositions of soil microbial communities (43).

Similar to the functional composition analysis, we found a significant tree mycorrhizal type effect on the functional diversity of soil microbial communities. Nonetheless, this effect was relatively weak and found only in monospecific stands. The results are in line with the significant effect of tree mycorrhizal type on the functional gene ortholog (GO) richness of fungi and bacteria as reported by Bahram et al. (44). We did not encounter any significant tree diversity effect on the functional diversity of soil microbial co-occurring communities, which was contrary to previous findings of the positive effects of plant diversity on microbial community functions and activities (71–73). Although this effect was not significant, we observed the tendency of increased microbial functional diversity under EcM trees in multi-tree-species mixtures. One might expect that the positive effect of tree diversity on the functional diversity of microbial communities might become significant in the long term (74, 75).

Moreover, our findings revealed that high tree diversity that includes both AM and EcM mycorrhizal type trees can harbor rich and converging functional genomic potential, which in turn, can have a positive effect on the studied ecosystem. This conforms to the previous findings of our study site of higher stand-level productivity in multi-tree-species mixtures compared to monospecific stands (76). Hence, our study warrants further research on the detailed mechanisms of how soil microbial communities contribute to the increased above-ground productivity in more-species-rich stands.

### Insights into the functional abundance differences of EcM and AM TSP soil co-occurring microbial subcommunities.

Furthermore, we investigated how EcM and AM TSP soil microbial subcommunities at each tree diversity level differ in their genomic functional abundances. The ordination coupled with the fitting of the significantly contributing enzymes showed for monospecific stands that all of the C-cycling and most of the P-cycling enzymes were diverging in opposite directions of the ordination. These C-cycling enzymes along with amidase and chitinase (N-cycling enzymes) might have similar functional roles in the community, which in this case could be the decomposition of complex carbohydrates for microbial utilization (77–79). In the other direction, the P-cycling enzymes were broadly involved in inorganic P solubilization and organic P mineralization, along with a set of N-cycling enzymes that take part in nitrification (e.g., hydroxylamine reductase) and nitrate reduction (e.g., ferredoxin-nitrite reductase). These findings indicate that these subcommunities might have major functional roles in producing plant- and microbe-available forms of N and P (79–81). This view was corroborated by the response of these modules to the soil chemistry as seen from dbRDA analysis. In contrast, in two-tree-species mixtures, a higher number of nutrient cycling enzymes did not show any distinct pattern, and this might indicate that the module differentiation was possibly driven by multiple functional differences. In multi-tree-species mixtures, fewer correlated enzymes were found, and this might reflect that the module differentiation was driven by fewer functional differences. Expectedly, P-cycle enzymes were predominantly correlated with the module differentiation at all tree diversity levels, and together with their relationship to soil nutrients in monospecific stands, suggests that the soil microbial subcommunities at our study site are shaped by the P limitation, which is in line with previous reports (57, 82, 83). Intriguingly, our subcommunity-level functional analysis pointed out the natural selection of microbes with required functional potential suitable to the habitat at community and subcommunity levels.

Furthermore, we encountered differences in functional abundances of nutrient cycling combinations at the module level among the EcM and AM TSP soil microbial communities. Overall, AM modules had a higher number of significantly abundant modules, except for C and N cycles. In particular, significantly abundant EcM modules for the C cycle were encountered more often in monospecific stands, while not a single significantly abundant EcM module was found in multi-tree-species mixtures. The higher abundance pattern in monospecific stands of such modules can be explained by the fact that ectomycorrhizal fungi can efficiently sequester carbon from plants (6, 84), influence the recruitment of co-occurring microbes, including bacteria (85, 86), and then can allocate the C to them (87–89). In support of this interpretation, we observed a major contribution of bacteria compared to fungi to the nutrient cycling potential in EcM modules in monospecific stands. In monospecific stands, for the N cycle, we found three significantly abundant EcM modules and one significantly abundant AM module. A recent soil metagenomics-based study from temperate forests (90) reported a larger estimated amount of N-cycling genes in AM than in EcM tree-dominated soils. In our study, we focused on those subcommunities that fulfill specific functional roles, which would explain the aforementioned observation. Nevertheless, in concordance, we found a relatively higher number of significantly abundant AM modules in two-tree-species mixtures. It is known that soils under AM trees have more open and faster nutrient cycling rates than EcM systems (6, 59), which is facilitated by the specifically associated fast-cycling versus slow-cycling microbes (91–93). In agreement with this assumption, we found an overall higher number of significantly abundant AM modules under the remaining nutrient cycling combinations (P, CN, CP, NP, and CNP).

Moreover, the number of modules that differed between EcM and AM was fewer in multi-tree-species stands compared to monospecific stands and two-tree-species mixtures. Taken together, these findings suggest converging genomic functional potential of EcM and AM soil microbiota at the subcommunity level with increasing tree species richness. Additionally, pairwise module analysis within tree mycorrhizal type resulted in a higher proportion of significant differences within AM subcommunities than that of EcM subcommunities in all nutrient cycling combinations, except for CNP, where equal proportions were observed. This might point to a higher functional equivalence in EcM subcommunities, which is probably facilitated by the slow-cycling members, such as ectomycorrhizal fungi, as reflected by members of the Agaricomycetes, which were the predominant differentially abundant fungal contributors to the nutrient cycling in monospecific stands and two-tree-species mixtures. In contrast, a higher number of specialized functional units in the AM subcommunities might be promoted by fast-cycling microbes, such as saprotrophs, which is reflected in their higher functional abundances in most of the nutrient cycling combinations and also by their differentially abundant taxa. Higher functional abundance in their subcommunities might confer resilience to the AM TSPs’ soil microbial communities. This expected functional resilience in AM and the functional equivalence in EcM TSP soil microbial communities can foster soil microbiome stability, which would be most pronounced in multi-tree-species mixtures (94).

### Differentially abundant taxa and the top contributors to the functional abundance and nutrient cycling combinations.

Finally, differential abundance analysis revealed the taxa behind the differences between each EcM and AM significantly soil-responsive module pairs within each tree diversity level. Agaricomycetes are a phylogenetically diverse group of fungi containing both biotrophs, such as ectomycorrhizal fungi and saprotrophs (95, 96), which explains their predominant contributions to the nutrient cycling combinations. Sordariomycetes were one of the major contributors to the nutrient cycling combinations in AM monospecific stands and also for both EcM and AM in multi-tree-species mixtures. Sordariomycetes are known to contain decomposers of wood and leaf litter (97, 98). A recent study identified some Sordariomycetes taxa to function as connector hubs in soil microbial networks and were positively correlated with the abundance of functional genes involved in C, N, and P cycling (99). Eurotiomycetes and Leotiomycetes, which contributed to various nutrient cycling combinations in our study, were also shown to have a significant link to the production of C-cycling enzymes (100). In addition, Eurotiomycetes were also found to be involved in denitrification (101). *Acidobacteria* and *Alphaproteobacteria* were the predominant contributors in all nutrient cycling combinations. Together with the *Actinobacteria*, which showed the second highest association with C in our study, all of these groups are known from the literature to be involved in the C cycle (100, 102), N cycle (90), and P cycle (12, 15). We have also shown the functional potential of these groups for other nutrient combinations, including CN, CP, NP, and CNP. This information can be helpful in future studies on the relationship between microbial taxa and nutrient cycling. Although these top differentially abundant classes were common in both EcM and AM modules, it is worth noting that they differ in their role at the lower taxon levels, such as ASVs. Moreover, the top two contributing fungal and bacterial classes differed between EcM and AM modules in the different tree diversity levels, especially in two-tree-species mixtures. This indicates that the subcommunities recruit groups of different taxa depending on their functional roles and niche requirements.

### Conclusions.

Taken together, our study highlights the importance of interkingdom soil microbial co-occurrence networks and their subcommunities to understand the factors that shape their community composition and functional roles. We comprehensively characterized the predicted genomic functional potential of co-occurring EcM and AM TSPs soil microbial subcommunities. Our analysis indicated that the nutrient cycling potential of the soil microbiota at the community level was a cumulative effect of their subcommunities. More importantly, functional potential differences, driven by differentially enriched taxa, were revealed among subcommunities that were not obvious at the community level. Our results highlight the key role of the tree mycorrhizal type in the recruitment and organization of these networks. Furthermore, higher tree diversity levels of coexisting AM and EcM mycorrhizal trees were found to foster microbial communities with rich and converging functional genomic potential, thereby promoting stable and better functioning of the forest soil ecosystem. These findings underline the versatility and significance of microbial subcommunities in different soil nutrient cycling processes, which contribute to maintaining multifunctionality and modulating tree-tree interactions in diverse forest ecosystems.

## MATERIALS AND METHODS

For detailed descriptions of the study site and design, sampling procedures, laboratory analyses and data generation, please refer to the 2021 study by Singavarapu et al. (43).

### Study site, experimental design, and sampling.

The BEF-China tree diversity experimental study site (site A) contains native subtropical tree species with a diversity gradient ranging from monospecific stands to 24-species mixtures (50). The experimental site was planted in 2009 in the Chinese subtropics (Xingangshan, Jiangxi Province, Southeast China [29.08 to 29.11°N, 117.90 to 117.93°E]) on a total area of 18.4 ha. The plots have a size of 25.8 m by 25.8 m, with 400 trees each spaced on a regular grid at 1.29 m. In our study design, two adjacent target trees were considered a tree-species pair (TSP) (103), and we focused on the conspecific TSPs, including six EcM and six AM type TSPs for this study. TSPs were randomly selected across 55 plots, with three replicates in each of the monospecific stands (denoted as “Mono”), two-tree-species mixtures (denoted as “Two”), and multi-tree-species mixtures (denoted as “Multi”), which comprised plots with a tree species richness of ≥4. This resulted in a total of 108 TSPs with the following six combinations: EcM|Mono (*n* = 18), EcM|Two (*n* = 18), EcM|Multi (*n* = 18), AM|Mono (*n* = 18), AM|Two (*n* = 18), and AM|Multi (*n* = 18). For more details on the study site, design and sampling, please refer to the 2021 study by Singavarapu et al. (43) (see Table S1 and Fig. S1 in the reference 43). Four soil cores (diameter of 5 cm and depth of 10 cm) were collected from the tree-tree interaction zone (i.e., the horizontal axis between the two partner trees of a TSP) at distances of 5 cm from the center of a TSP (first two cores) and a further 20 cm away (other two cores). A composite soil sample was made from the four soil cores after pooling, mixing, and removal of root fragments by sieving the mixed soil through a 2-mm-pore mesh-size sieve. Soil samples for microbiota analyses (30 g) were freeze-dried (104) and stored at −80°C until further analyses.

### Soil characteristics.

Soil samples were divided into two parts for the measurement of soil moisture and other soil variables. Soil moisture was measured by drying the soil at 105°C for 24 h. Soil pH was measured in a 1:2.5 soil-water solution with a Thermo Scientific Orion Star A221 pH meter after air drying of the soil at 40°C for 2 days. Soil total organic carbon (TOC) was measured using a TOC analyzer (Liqui TOC II; Elementar Analysensysteme GmbH, Hanau, Germany). Soil total nitrogen (TN) was measured using an autoanalyzer (SEAL Analytical GmbH, Norderstedt, Germany) by the Kjeldahl method (105). Soil total phosphorus (TP) was measured following wet digestion with H_2_SO_4_ and HClO_4_ using a UV-visible (UV-Vis) spectrophotometer (UV2700; Shimadzu, Japan). NH_4_^+^ and NO_3_^−^ were measured using the colorimetric method with a Smart Chem 200 Discrete auto analyzer (AMS, Italy) after extraction with 2 M KCl (106).

### Sequencing of microbial communities.

Briefly, soil microbial genomic DNA was extracted using PowerSoil DNA isolation kit (Mo Bio Laboratories, Inc., Carlsbad, CA, USA), followed by quantification using a NanoDrop spectrophotometer (Thermo Fisher Scientific, Dreieich, Germany). The bacterial amplicon libraries were prepared by the amplification of the V4 region of the bacterial 16S rRNA gene using the universal primer pair 515f and 806r (107) with Illumina adapter sequence overhangs. Fungal amplicon libraries were prepared by seminested PCR, first to amplify the internal transcribed spacer 2 (ITS2) ribosomal DNA (rDNA) region using the ITS1F (108) and ITS4 (109) primers, followed by a second amplification round with the primer pair fITS7 (110) and ITS4 containing the Illumina adapter sequences. Both amplicon libraries were purified with AMPure XP beads (Beckman Coulter, Krefeld, Germany), and then Illumina Nextera XT indices were added to those libraries using the indexing PCR, followed by another round of purification with AMPure XP beads. The indexed amplicon libraries were quantified by PicoGreen assay and then pooled equimolarly to a final concentration of 4 nM each for fungi and bacteria. Furthermore, the final library with the pool of fungal and bacterial libraries was sequenced (paired-end sequencing of 2 × 300 bp with MiSeq reagent kit v.3) on an Illumina MiSeq platform (Illumina, Inc., San Diego, CA, USA) at the Department of Environmental Microbiology, UFZ, Leipzig, Germany.

### Bioinformatics analysis.

Bioinformatics analysis was performed using the Quantitative Insights into Microbial Ecology (QIIME 2 2020.2) (111) software. Raw reads were demultiplexed, and primer sequences were trimmed, followed by sequence denoising and grouping into amplicon sequence variants (ASVs) using cut-adapt (112) (q2-cutadapt) and DADA2 (113) (q2-dada2), respectively. Taxonomy assignment was made using the q2-feature-classifier (114) with a classify-sklearn naive Bayes taxonomy classifier against the silva-132-99-515-806-nb-classifier and unite-ver8-99-classifier-04.02.2020 for bacteria and fungi, respectively. The resulting fungal and bacterial ASV matrices, taxonomic tables, and representative sequences were transferred to R software (v.4.0.2) using the phyloseq package (115). The ASV matrices were rarefied to 16,542 and 28,897 reads per sample, for fungi and bacteria, respectively, to control for differential sequencing depths. To identify the microbial taxa that are faithfully represented in each of the tree mycorrhizal type and tree diversity combinations (*viz*., EcM|Mono, EcM|Two, EcM|Multi, AM|Mono, AM|Two, and AM|Multi), stringent filtering steps were applied to fungal and bacterial data sets prior to further data analyses. First, all taxa with an abundance of >3% mean total sequencing reads were filtered, resulting in 798 bacterial and 728 fungal taxa. Next, in each of the tree mycorrhizal type and tree diversity combinations, the taxa were further filtered with a frequency of presence in at least 2/3 of the samples (≥33%) in their respective data sets. These filtered data sets from each combination were merged into one bacterial and one fungal data set each and were used as input into PICRUSt2 (Phylogenetic Investigation of Communities by Reconstruction of Unobserved States) software for the prediction of metagenome functional abundances (52).

In PICRUSt2, briefly, first, the ASV representative sequences of bacteria and fungi were multiple aligned with the 16S and ITS reference genome database files using hidden Markov models (HMMER tool). For bacteria, we used default settings, and for fungi, we used the minimum-alignment option of 0.5 (default 0.8) to include all of the taxa that were classified until the genus level in the output. Then these aligned sequences were placed into the reference phylogenetic tree constructed by the maximum likelihood phylogenetic placement method using EPA-ng (116) and Gappa tools (117). Next, gene family content was predicted for both bacterial and fungal ASVs based on EC (Enzyme Commission/Classification) numbers (118) using the castor package (119). Here, we filtered the predicted EC content tables of bacteria and fungi for the carbon, nitrogen, and phosphorus nutrient cycling-related EC numbers (enzymes) based on previously available literature (Table S3). Finally, these filtered EC content tables were used to determine the gene family abundances per sample with respect to nutrient cycling for both bacterial and fungal data sets. Here, one ASV in each bacterial and fungal data set was removed as they were above the default NSTI (nearest-sequenced-taxon index) values, the metric that identifies the ASVs that are far from all the reference sequences, thus allowing us to exclude less reliable predictions.

### Statistical analysis.

All of the statistical analyses were done in R (version 4.0.2) software. EcM and AM TSPs’ soil bacterial and fungal interkingdom co-occurrence networks were constructed at each tree diversity level (*viz.*, EcM|Mono, EcM|Two, EcM|Multi, AM|Mono, AM|Two, and AM|Multi) using the filtered data sets (i.e., [i] an abundance of >3% mean total sequencing reads and [ii] present in at least 2/3 of the samples) mentioned in the bioinformatics analysis. Networks were constructed using the R package SpiecEasi (120). SpiecEasi controls the spurious co-occurrences by controlling for the lack of independence in normalized count data, which accounts for the high number of edges in the network-based analysis of amplicon data sets. Networks were estimated by the Meinshausen and Bühlmann graph inference method. The minimum λ ratio was 10^−3^, and network assessment was done over 100 values of λ for every 50 cross-validations. Network structural and topological properties, including edges, centrality indices, modularity, etc., were calculated using the igraph package (121). Modules that are considered to be subcommunities in each network were determined based on a hierarchical agglomeration algorithm with modularity optimization using the “cluster_fast_greedy” function. Differences in the distribution of four network centrality measures (degree, betweenness, closeness, and eigen centralities) between EcM and AM TSPs’ soil microbial networks were tested by bootstrapping with 10,000 iterations, followed by a two-sample Kolmogorov-Smirnov test using the ‘ks.test’ function in R. Furthermore, these distributions were visualized with sinaplots using the ggforce and ggplot2 packages. Network modules that were significantly associated with soil chemical properties were determined using dbRDA (distance-based redundancy analysis) models based on the Bray-Curtis distance using the “capscale” function in the vegan package (122), and for this, modules with a size of ≥40 were considered. Soil variables (C, N, P, C/N, C/P, N/P, TOC, SOM, NH_4_^+^, NO_3_^−^, pH, and moisture) were standardized to a mean of zero and standard deviation of 1 (“decostand” function in vegan). Multicolinearity was checked using the “vifstep” function in the usdm package (123). Furthermore, important soil variables were selected using stepwise model selection (the “ordistep” function in vegan), and the variables selected were included in the final model for each subcommunity. Variables that were significant in the final model were considered the significant soil characteristics, and the subcommunities that were associated with at least one of these significant soil variables were treated as soil-responsive subcommunities, in the following called “significant” modules.

The predicted gene family abundance matrices from PICRUSt2 output were merged per EC number to yield the co-occurring community enzyme/gene family abundance (functional abundance) matrices. These functional compositions were categorized into nutrient cycling combinations (C, N, P, CN, CP, NP, and CNP) based on the constituent EC numbers. Shannon diversity of these functional abundance matrices was calculated as a measure for functional diversity and tested for the effects of tree diversity and tree mycorrhizal type using two-way analysis of variance (ANOVA) with the “aov” function in R. Furthermore, within each tree diversity level, pairwise comparison of tree mycorrhizal type was done with *t* tests followed by Benjamini-Hochberg (BH) multiple testing correction. The effects of the tree diversity and tree mycorrhizal type on the functional compositions were tested with Bray-Curtis distance-based permutational multivariate analysis of variance (PERMANOVA) using the vegan package. Moreover, the functional composition of the whole community was compared with those of the soil-responsive modules, and consequently, all the analyses based on subcommunities were rerun using only the soil-responsive subcommunities.

To derive subcommunity relative functional abundances, first, mean taxon relative abundances of subcommunities in each network were calculated using the normalized bacterial and fungal ASV abundances from the PICRUSt2 output. Next, matrix multiplication was applied using the mean taxon relative abundances of subcommunities and the predicted EC content (gene family numbers) matrix of the taxa as shown in the exemplary formula shown in equation 1. In equation 1, the matrix on the left-hand side is a matrix of module (mod1, mod2) by taxon (t1, t2, t3) with the taxon’s mean relative abundances in the modules, and the one on the right-hand side is a matrix of taxon (t1, t2, t3) by enzyme (e1, e2), with the number of enzyme gene families per taxon. The result is a matrix with gene family abundances of enzymes (i.e., functional abundances) in each module (mod1, mod2).

(1) $$\begin{array}{c} \text{mod1}\\ \text{mod2} \end{array}\overset{\text{t1 t2 t3 }}{\left[\begin{array}{c} 0.10\;0.19\;0.07\\ 0.02\;0.03\;0.06 \end{array}\right]}\;\times \;\overset{\text{ e1 e2}}{\begin{array}{c} \text{t1}\\ \text{t2}\\ \text{t3} \end{array}\left[\begin{array}{c} 1 7\\ 2 1\\ 4 3 \end{array}\right]}\;=\;\begin{array}{c} \text{mod1}\\ \text{mod2} \end{array}\overset{\text{e1 e2 }}{\left[\begin{array}{c} 0.76\;1.10\\ 0.32\;0.35 \end{array}\right]}$$

The obtained subcommunity functional abundances across tree diversity levels were visualized by ordination with PCoA, using the ape package (124). Moreover, enzymes related to C, N, and P cycling were fitted to the ordination using “envfit” function in Vegan. Those enzymes with a *P* value of <0.01 were considered significantly associated with the differentiation of modules. Furthermore, pairwise comparisons of subcommunity functional abundances at each tree diversity level were done with Wilcoxon signed-rank tests followed by BH multiple-testing correction with a significance threshold of *P* < 0.01 using the rstatix package, and the results are presented as a heat map using ComplexHeatmap package (125). In addition, taxon differential abundance tests were performed for all EcM and AM modules that were significantly different on the overall CNP relative functional abundance of each ASV per subcommunity. The latter was obtained by multiplying the relative abundance of that ASV by its predicted EC content. Pairwise Wilcoxon rank sum tests (BH multiple-testing correction with a significance threshold of *P* < 0.01) were used to determine the differentially abundant ASVs between subcommunity pairs and aggregated these significant ASVs at the class taxonomic level. The relative functional abundance proportions of the top two of each of the fungal and bacterial classes per tree diversity level in subcommunities of each of the EcM and AM TSPs’ soil microbial networks were visualized as Sankey diagrams using the networkD3 package (126).

### Data availability.

The data sets generated for this study can be found in the National Center for Biotechnology Information (NCBI) Sequence Read Archive (SRA) under BioProject no. PRJNA702024.
