# Supplementary material for: Functional Potential of Soil Microbial Communities and Their Subcommunities Varies with Tree Mycorrhizal Type and Tree Diversity
Source: Microbiol Spectr. 2023 Mar 23;11(2):e04578-22. doi: 10.1128/spectrum.04578-22 (PMC10111882; doi:10.1128/spectrum.04578-22)
Supplement: Supplemental file 1 — Supplemental material. Download spectrum.04578-22-s0001.pdf, PDF file, 0.6 MB [file spectrum.04578-22-s0001.pdf]

Supplementary Material

The functional potential of soil microbial communities and their sub-communities varies with tree mycorrhizal type and tree diversity

Bala Singavarapu, Jianqing Du, Rémy Beugnon, Simone Cesarz, Nico Eisenhauer, Kai Xue, Yanfen Wang, Helge Bruelheide & Tesfaye Wubet

Table S1

Network properties of EcM and AM TSPs soil co-occurring microbial communities across the tree diversity levels

	Input Bacteria	Input Fungi	Nodes	Edges negative	Edges positive	Average path length	Clustering coefficient	Modularity	Modules	Diameter
AM | Mono	798	473	1271	8180	9486	2.585	0.085	0.208	5	5
AM | Two	798	503	1301	8048	10124	2.587	0.087	0.214	6	5
AM | Multi	797	491	1288	7848	9670	2.602	0.083	0.211	8	5
EcM | Mono	796	448	1244	7042	8867	2.671	0.094	0.236	8	6
EcM | Two	798	430	1228	7260	8538	2.638	0.083	0.210	9	5
EcM | Multi	798	514	1312	8114	9703	2.634	0.091	0.222	7	5

The functional potential of soil microbial communities and their sub-communities varies with tree mycorrhizal type and tree diversity

Bala Singavarapu, Jianqing Du, Rémy Beugnon, Simone Cesarz, Nico Eisenhauer, Kai Xue, Yanfen Wang, Helge Bruelheide & Tesfaye Wubet

Table S2

Significant soil variables associated with EcM and AM TSPs soil microbial network sub-communities

Network	*Module*	*Size*	*pH*		*NO3*		*Moisture*		*TOC*		*P*		*N*		*NH4*	
			F	*P*	*F*	*P*	*F*	*P*	*F*	*P*	*F*	*P*	*F*	*P*	*F*	*P*
AM | Mono	1	355	1.91	0.004	-	-	-	-	1.56	0.031	2.13	0.003	-	-	-	-
	2	88	4.38	0.001	2.11	0.026	-	-	-	-	2.20	0.014	-	-	-	-
	3	418	8.53	0.001	-	-	-	-	-	-	1.78	0.012	-	-	-	-
	5	403	2.79	0.003	-	-	-	-	-	-	2.15	0.014	-	-	-	-
AM | Two	1	363	5.90	0.001	-	-	-	-	-	-	-	-	-	-	-	-
	2	419	2.09	0.004	1.51	0.04	-	-	-	-	-	-	-	-	-	-
	3	450	2.68	0.001	-	-	-	-	-	-	-	-	2.55	0.002	-	-
	5	40	-	-	-	-	-	-	-	-	-	-	-	-	2.34	0.02
AM | Multi	2	434	3.17	0.006	-	-	2.27	0.027	-	-	-	-	-	-	-	-
	3	403	1.73	0.009	1.51	0.01	-	-	-	-	-	-	-	-	-	-
	8	372	4.75	0.001	-	-	2.12	0.041	-	-	-	-	-	-	-	-
EcM | Mono	3	414	1.40	0.083	-	-	-	-	1.51	0.042	-	-	-	-	-	-
	4	351	1.62	0.037	1.57	0.035	-	-	-	-	-	-	-	-	-	-
	8	388	3.19	0.003	-	-	-	-	-	-	2.09	0.042	-	-	-	-
EcM | Two	1	82	5.61	0.001	2.42	0.03	-	-	-	-	-	-	-	-	3.13	0.008
	2	373	4.02	0.001	2.88	0.003	-	-	-	-	-	-	-	-	2.03	0.023
	8	303	3.60	0.001	2.21	0.01	-	-	-	-	-	-	-	-	2.77	0.004
	9	411	2.74	0.002	1.93	0.011	-	-	-	-	-	-	-	-	-	-
EcM | Multi	1	340	6.81	0.001	-	-	-	-	-	-	-	-	-	-	-	-
	4	433	1.98	0.028	-	-	-	-	-	-	-	-	-	-	-	-
	7	437	2.39	0.002	-	-	-	-	-	-	-	-	-	-	-	-

Significant soil parameters were selected based on dbRDA models for each network. Variables that were significant only ($p < 0.05$) in the final model were shown here. Fields with '-' indicate the non-significance of the variable in that particular category.

The functional potential of soil microbial communities and their sub-communities varies with tree mycorrhizal type and tree diversity

Bala Singavarapu, Jianqing Du, Rémy Beugnon, Simone Cesarz, Nico Eisenhauer, Kai Xue, Yanfen Wang, Helge Bruelheide & Tesfaye Wubet

Table S3

EC (Enzyme Commission/Classification) numbers of the enzymes known to participate in C, N and P cycles based on the literature

S.No	Name	EC	Description	Nutrient cycle	Reference
1	Beta-glucosidase	EC:3.2.1.21	Cleaving of cellobiose to free glucose molecules by hydrolysis of β-glucosidic linkages	Carbon	Zang et al. (2018); López-Mondéjar et al. (2016); Canarini et al. (2021)
2	Exoglucanase	EC:3.2.1.91	*CBH1; CBH2 [K19668];* cellulose 1,4-beta-cellobiosidase; exo-cellobiohydrolase;	Carbon	Canarini et al. (2021)
3	Endoglucanase	EC:3.2.1.4	Also known as Cellulase [K19357]; *bcsZ [*K20542*]*	Carbon	Das, S. K., & Varma, A. (2010); Zang et al. (2018);López-Mondéjar et al. (2016)
4	xylan 1,4-betaxylosidase	EC:3.2.1.37	Degradation of polysaccharide xylan into xylose. Catalysing the hydrolysis of the glycosidic linkage (β-1,4) of xylosides	Carbon	Zang et al. (2018);López-Mondéjar et al. (2016); Canarini et al. (2021)
5	Endo-1,4-betaxylanase	EC:3.2.1.8	Degradation of polysaccharide xylan into xylose. Catalysing the hydrolysis of the glycosidic linkage (β-1,4) of xylosides	Carbon	Zang et al. (2018);López-Mondéjar et al. (2016)
6	Laccase	EC:1.10.3.2	lccA; Other KO is K05909. Also known as benzenediol:oxygen oxidoreductase. A group of multi-copper proteins of low specificity acting on both o- and p-quinols, and often acting also on aminophenols and phenylenediamine.	Carbon	Das, S. K., & Varma, A. (2010);Zang et al. (2018);López-Mondéjar et al. (2016)
7	Pectin lyase	EC:4.2.2.10	PL; Other KO is K05909. Also known as pectolyase and polymethylgalacturonic transeliminase.	Carbon	Das, S. K., & Varma, A. (2010)
8	Peroxidase	EC:1.11.1.7	Oxidoreductases; Acting on a peroxide as acceptor. Other KO is K19511.	Carbon	Das, S. K., & Varma, A. (2010);Zang et al.

					(2018);López-Mondéjar et al. (2016)
9	Alpha-N-acetylglucosaminidase	EC:3.2.1.50	NAGLU; Hydrolysis of terminal non-reducing N-acetyl-D-glucosamine residues in N-acetyl-alpha-D-glucosaminides	Carbon	Das, S. K., & Varma, A. (2010); López-Mondéjar et al. (2016)
10	Alpha-amylase	EC:3.2.1.1	AMY, amyA, malS; Endohydrolysis of (1->4)-alpha-D-glucosidic linkages in polysaccharides containing three or more (1->4)-alpha-linked D-glucose units	Carbon	Das, S. K., & Varma, A. (2010); Zang et al. (2018)
11	Triacylglycerol lipase	EC:3.1.1.3	triacylglycerol acylhydrolase; Glycerolipid metabolism	Carbon	Canarini et al. (2021)
12	Nitrogenase	EC:1.18.6.1	*anfG [K00531]; nifD[K02586]; nifK* [K02591]; ;Nitrogenase, the enzyme complex catalysing N2 fixation. Reduced ferredoxin:dinitrogen oxidoreductase (ATPhydrolysing)	Nitrogen	Pajares and Bohannan (2016); Kuypers et al. (2018); Black et al. (2018)
13	Ammonia monooxygenase subunit A	EC:1.14.99.39	*AMO [K10944-K10946]*; Conversion of N into usable forms by oxidation. The enzyme catalyses the first reaction in the pathway of ammonia oxidation to nitrite	Nitrogen	Pajares and Bohannan (2016); Kuypers et al. (2018); Isobe et al. (2020); Black et al. (2018)
14	Urease	EC:3.5.1.5	URE; Also known as urea amidohydrolase. *ureA, ureB, ureC* (urease subunit gamma, beta & alpha) [K01428 - K01430, K14048]	Nitrogen	Kuypers et al. (2018); Isobe et al. (2020); Black et al. (2018)
15	Hydroxylamine reductase	EC:1.7.99.1	This enzyme participates in nitrogen metabolism acting on other nitrogenous compounds as donors with a cytochrome as an acceptor	Nitrogen	Pajares and Bohannan (2016); Kuypers et al. (2018)
16	Nitrite reductase / Hydroxylamine reductase	EC:1.7.2.1	*nirK [K00368] ;nirS [K15864]*; Also known as nitric-oxide:ferricytochrome-c oxidoreductase.	Nitrogen	Pajares and Bohannan (2016); Kuypers et al. (2018) ; Black et al. (2018)
17	Hydrazine synthase	EC:1.7.2.7	*HZS [K20932-K20934]*; anaerobic ammonium oxidation	Nitrogen	Black et al. (2018)
18	Hydrazine dehydrogenase	EC:1.7.2.8	*hdh*; anaerobic ammonium oxidation	Nitrogen	Black et al. (2018)
19	Nitrous-oxide reductase	EC:1.7.2.4	*nosZ*; N2O reductase;	Nitrogen	Black et al. (2018)

20	Nitric oxide reductase	EC:1.7.2.5	*norB*; nitric oxide reductase subunit B. nitric oxide reductase (cytochrome c)	Nitrogen	Black et al. (2018)
21	Ferredoxin-nitrite reductase	EC:1.7.7.1	*nirA*; ferredoxin-nitrite reductase. Also known as ammonia:ferredoxin oxidoreductase	Nitrogen	Black et al. (2018)
22	Ferredoxin-nitrate reductase	EC:1.7.7.2	*narB*; assimilatory ferredoxin-nitrate reductase	Nitrogen	Black et al. (2018)
23	Nitrate reductase	EC:1.7.5.1	*narG, narZ, nxrA; narV; NarGHI; [K00370, K00371, K00374]* Dissimilatory nitrate reductase	Nitrogen	Black et al. (2018)
24	Periplasmic nitrate reductase	EC:1.9.6.1	*napA; napB[K02568];*respiratory nitrate reductase; nitrate reductase (cytochrome);	Nitrogen	Black et al. (2018)
25	Hydroxylamine dehydrogenase	EC:1.7.2.6	*hao;* The enzyme converts hydroxylamine to nitrite	Nitrogen	Black et al. (2018)
26	Nitrite reductase (NADH)	EC:1.7.1.15	*nirB;* NADH large subunit. *nirD [K00363];* NADH small subunit	Nitrogen	Black et al. (2018)
27	Nitrite reductase (cytochrome c-552)	EC:1.7.2.2	*nrfA;*	Nitrogen	Black et al. (2018)
28	Chitinase	EC:3.2.1.14	ChiC; The enzyme binds to chitin and randomly cleaves glycosidic linkages in chitin and chitodextrins in a non-processive mode, generating chitooligosaccharides and free ends on which exo-chitinases and exo-chitodextrinases can act.	Nitrogen	Canarini et al. (2021)
29	Endo-chitodextinase	EC:3.2.1.202	endo_I; Also known as chitodextrinase.	Nitrogen	Canarini et al. (2021)
30	Exo-chitinase (reducing end)	EC:3.2.1.201	ChiA; The enzyme hydrolyses the second glycosidic (1->4) linkage from reducing ends of chitin and chitodextrin molecules, liberating N,N'-diacetylchitobiose disaccharides	Nitrogen	Canarini et al. (2021)
31	Exo-chitinase (non-reducing end)	EC:3.2.1.200	ChiB; The enzyme hydrolyses the second glycosidic (1->4) linkage from reducing ends of chitin and chitodextrin molecules, liberating N,N'-diacetylchitobiose disaccharides	Nitrogen	Canarini et al. (2021)
32	Endo-beta-N-acetylglucosaminidase	EC:3.2.1.96	ENGASE; Glycosidases, i.e. enzymes that hydrolyse O- and S-glycosyl compounds.Endohydrolysis of the N,N'-diacetylchitobiosyl unit in high-mannose glycopeptides and glycoproteins	Nitrogen	Canarini et al. (2021); Isobe et al. (2020)

33	Leucyl aminopeptidase	EC:3.4.11.1	CARP, pepA; Also known as leucine aminopeptidase and peptidase S. Release of an N-terminal amino acid. Amino acid amides and methyl esters are also readily hydrolysed, but rates on arylamides are exceedingly low	Nitrogen	Canarini et al. (2021)
34	Aminopeptidase N	EC:3.4.11.2	pepN; Also known as aminopeptidase M and alanine aminopeptidase. Release of an N-terminal amino acid, Xaa!Yaa- from a peptide, amide or arylamide.	Nitrogen	Canarini et al. (2021)
35	Amidase	EC:3.5.1.4	amiE; Also known as acylamidase and acylamide amidohydrolase. Acting on carbon-nitrogen bonds, other than peptide bonds; In linear amides	Nitrogen	Das, S. K., & Varma, A. (2010)
36	Acid phosphatase	EC:3.1.3.2	*olpA;* PHO;Transformation of P from soil organic matter into available forms. Hydrolytic enzymes that cleave the ester bond between the phosphate group and the organic residue of the organic phosphates	Phosphorus	Eivazi and Tabatabai (1977); Margalef et al. (2017)
37	Inorganic Pyrophosphatase	EC:3.6.1.1	*ppa*; Also known as diphosphate phosphohydrolase	Phosphorus	Eivazi and Tabatabai (1977); Margalef et al. (2017)
38	Exopolyphosphatase	EC:3.6.1.11	*ppx;* Also known as guanosine-5'-triphosphate,3'-diphosphate pyrophosphatase	Phosphorus	Dai et al. (2020)
39	phosphoribosyl 1,2-cyclic phosphate phosphodiesterase	EC:3.1.4.55	*phnP*; C-P lyase sub-unit	Phosphorus	Gaiero et al. (2021)
40	Alpha-D-ribose 1-methylphosphonate 5-triphosphate diphosphatase	EC:3.6.1.63	*phnM*; C-P lyase sub-unit	Phosphorus	Dai et al. (2020)
41	Alpha-D-ribose 1-methylphosphonate 5-phosphate C-P lyase	EC:4.7.1.1	*phnJ*; C-P lyase sub-unit. Participates in processing of phophonates into usable phosphate	Phosphorus	Gaiero et al. (2021)
42	Alpha-D-ribose 1-methylphosphonate 5-triphosphate synthase	EC:2.7.8.37	*phnI*; C-P lyase sub-unit. ; *phnH* ; *phnG* ; *phnL*	Phosphorus	Dai et al. (2020)
43	Ribose 1,5-bisphosphokinase	EC:2.7.4.23	*phnN*; C-P lyase sub-unit.	Phosphorus	Dai et al. (2020)

44	Aminoalkylphosphonate N-acetyltransferase	EC:2.3.1.280	phnO;	Phosphorus	Dai et al. (2020)
45	Quinoprotein glucose dehydrogenase	EC:1.1.5.2	*gcd; PQQ*;	Phosphorus	Dai et al. (2020)
46	Phosphonoacetaldehyde hydrolase	EC:3.11.1.1	*phnX ;* Also known as phosphonatase	Phosphorus	Gaiero et al. (2021)
47	2-aminoethylphosphonate-pyruvate transaminase	EC:2.6.1.37	*phnW;*	Phosphorus	Dai et al. (2020)
48	Phosphonoacetate hydrolase	EC:3.11.1.2	*phnA*; A zinc-dependent enzyme. Belongs to the alkaline phosphatase superfamily of zinc-dependent hydrolases.	Phosphorus	Gaiero et al. (2021)
49	Inositol-phosphate phosphatase	EC:3.1.3.25	IMPA; suhB; Also known as myo-inositol-1(or 4)-monophosphatase. Acts on five of the six isomers of myo-inositol phosphate, all except myo-inositol 2-phosphate,	Phosphorus	Gaiero et al. (2021)
50	Alkaline phosphatase	EC:3.1.3.1	*phoA, phoB;* Also known as phosphate-monoester phosphohydrolase. Wide specificity. Also catalyses transphosphorylations. ; *phoD*	Phosphorus	Gaiero et al. (2021)
51	3-phytase	EC:3.1.3.8	*phy;* Also known as myo-inositol-hexakisphosphate 3-phosphohydrolase	Phosphorus	Liang et al. (2020)
52	4-phytase	EC:3.1.3.26	*appA*; Also known as myo-inositol-hexakisphosphate 4-phosphohydrolase.Inositol phosphate metabolism	Phosphorus	Gaiero et al. (2021)
53	Glycerophosphoryl diester phosphodiesterase	EC:3.1.4.46	*glpQ, ugpQ;* Phosphoric-diester hydrolases. Glycerophospholipid metabolism.Broad specificity for glycerophosphodiesters	Phosphorus	Gaiero et al. (2021)
54	Phosphate regulon sensor histidine kinase	EC:2.7.13.3	*PhoR* ; Also known as histidine kinase. Two-component system. Transferring phosphorus-containing groups	Phosphorus	Gaiero et al. (2021)
55	Glycerol 3-phosphate transport system ATP-binding protein	EC:7.6.2.10	*ugpC*; Also known as ABC-type glycerol 3-phosphate transporter and ATP phosphohydrolase.Linked to the hydrolysis of a nucleoside triphosphate.	Phosphorus	Gaiero et al. (2021)

56	Phosphonate transport system ATP-binding protein	EC:7.3.2.2	*phnC*; ABC-type phosphonate transporter. Linked to the hydrolysis of a nucleoside triphosphate. Also known as phosphonate-transporting ATPase	Phosphorus	Gaiero et al. (2021)
57	Phosphate transport system ATP-binding protein	EC:7.3.2.1	*pstB*; ABC-type phosphate transporter. Also known as phosphate-transporting ATPase	Phosphorus	Gaiero et al. (2021)

The functional potential of soil microbial communities and their sub-communities varies with tree mycorrhizal type and tree diversity

Bala Singavarapu, Jianqing Du, Rémy Beugnon, Simone Cesarz, Nico Eisenhauer, Kai Xue, Yanfen Wang, Helge Bruelheide & Tesfaye Wubet

Table S4
List of PICRUSt2 predicted unique gene families/Enzymes in the EcM and AM TSPs soil co-occurring microbial communities

EC number	Name	Symbol	Nutrient Cycle
EC:3.1.1.3	*Triacylglycerol lipase*	*tagl*	*Carbon*
EC:3.2.1.1	*Alpha-amylase*	*AMY*	*Carbon*
EC:3.2.1.21	*Beta-glucosidase*	*bglX*	*Carbon*
EC:3.2.1.37	*Xylan 1.4-beta-xylosidase*	*xynB*	*Carbon*
EC:3.2.1.4	*Cellulase*	*CELB*	*Carbon*
EC:3.2.1.50	*Alpha-N-acetylglucosaminidase*	*NAGLU*	*Carbon*
EC:3.2.1.8	*Endo-1.4-beta-xylanase*	*xynA*	*Carbon*
EC:3.2.1.91	*Cellulose 1. 4-beta-cellobiosidase (non-reducing end)*	*CBH1*	*Carbon*
EC:4.2.2.10	*Pectin lyase*	*PL*	*Carbon*
EC:1.10.3.2	*Laccase*	*lccA*	*Carbon*
EC:1.11.1.7	*Peroxidase*	*PO*	*Carbon*
EC:1.14.99.39	*Ammonia monooxygenase*	*AMO*	*Nitrogen*
EC:1.18.6.1	*Nitrogenase*	*anfG*	*Nitrogen*
EC:1.7.1.15	*Nitrite reductase (NADH)*	*nirB*	*Nitrogen*
EC:1.7.2.1	*Nitrite reductase (NO-forming)*	*nirK*	*Nitrogen*
EC:1.7.2.2	*Nitrite reductase (cytochrome; ammonia-forming)*	*nrfA*	*Nitrogen*
EC:1.7.2.4	*Nitrous-oxide reductase*	*nosZ*	*Nitrogen*
EC:1.7.2.5	*Nitric-oxide reductase (cytochrome c)*	*norB*	*Nitrogen*
EC:1.7.2.6	*Hydroxylamine dehydrogenase*	*haoA*	*Nitrogen*
EC:1.7.7.1	*Ferredoxin--nitrite reductase*	*nirA*	*Nitrogen*
EC:1.7.7.2	*Ferredoxin--nitrate reductase*	*narB*	*Nitrogen*
EC:1.7.99.1	*Hydroxylamine reductase*	*HAO*	*Nitrogen*
EC:3.2.1.14	*Chitinase*	*ChiC*	*Nitrogen*
EC:3.4.11.1	*Leucyl aminopeptidase*	*pepA*	*Nitrogen*
EC:3.4.11.2	*Membrane alanyl aminopeptidase*	*pepN*	*Nitrogen*
EC:3.5.1.4	*Amidase*	*amiE*	*Nitrogen*
EC:3.5.1.5	*Urease*	*URE*	*Nitrogen*

EC:1.1.5.2	*Quinoprotein glucose dehydrogenase*	*PQQ*	*Phosphorus*
EC:2.6.1.37	*2-aminoethylphosphonate--pyruvate transaminase*	*phnW*	*Phosphorus*
EC:2.7.13.3	*Histidine kinase*	*PhoR*	*Phosphorus*
EC:2.7.4.23	*Ribose 1.5-bisphosphate phosphokinase*	*phnN*	*Phosphorus*
EC:2.7.8.37	*Alpha-D-ribose 1-methylphosphonate 5-triphosphate synthase*	*PhnI*	*Phosphorus*
EC:3.1.3.1	*Alkaline phosphatase*	*phoA*	*Phosphorus*
EC:3.1.3.2	*Acid phosphatase*	*PHO*	*Phosphorus*
EC:3.1.3.25	*Inositol-phosphate phosphatase*	*IMPA*	*Phosphorus*
EC:3.1.3.26	*4-phytase*	*appA*	*Phosphorus*
EC:3.1.3.8	*3-phytase*	*phy*	*Phosphorus*
EC:3.1.4.46	*Glycerophosphodiester phosphodiesterase*	*glpQ*	*Phosphorus*
EC:3.1.4.55	*Phosphoribosyl 1.2-cyclic phosphate phosphodiesterase*	*phnP*	*Phosphorus*
EC:3.11.1.1	*Phosphonoacetaldehyde hydrolase*	*phnX*	*Phosphorus*
EC:3.11.1.2	*Phosphonoacetate hydrolase*	*phnA*	*Phosphorus*
EC:3.6.1.1	*Inorganic diphosphatase*	*ppa*	*Phosphorus*
EC:3.6.1.11	*Exopolyphosphatase*	*ppx*	*Phosphorus*
EC:3.6.1.63	*Alpha-D-ribose 1-methylphosphonate 5-triphosphate diphosphatase*	*phnM*	*Phosphorus*
EC:4.7.1.1	*Alpha-D-ribose 1-methylphosphonate 5-phosphate C-P-lyase*	*phnJ*	*Phosphorus*

The functional potential of soil microbial communities and their sub-communities varies with tree mycorrhizal type and tree diversity

Bala Singavarapu, Jianqing Du, Rémy Beugnon, Simone Cesarz, Nico Eisenhauer, Kai Xue, Yanfen Wang, Helge Bruelheide & Tesfaye Wubet

Table S5
Two-way-ANOVA effects of tree mycorrhizal type and tree diversity on nutrient cycling functional diversity of the soil co-occurring fungal and bacterial communities

Nutrient Cycle	Factor	df	F	pval.adj
C	Mycorrhizal_Type (M)	1	2.121	0.24
	Tree_Diversity (L)	2	0.073	0.93
	Interaction (MxL)	2	0.758	0.55
N	Mycorrhizal_Type (M)	1	3.627	0.124
	Tree_Diversity (L)	2	1.011	0.482
	Interaction (MxL)	2	3.991	0.077
P	Mycorrhizal_Type (M)	1	9.826	**0.022***
	Tree_Diversity (L)	2	1.735	0.272
	Interaction (MxL)	2	2.136	0.216
CN	Mycorrhizal_Type (M)	1	5.42	0.077
	Tree_Diversity (L)	2	0.307	0.773
	Interaction (MxL)	2	2.974	0.124
CP	Mycorrhizal_Type (M)	1	11.056	**0.022***
	Tree_Diversity (L)	2	0.698	0.552
	Interaction (MxL)	2	2.808	0.124
NP	Mycorrhizal_Type (M)	1	7.4	**0.04***
	Tree_Diversity (L)	2	1.522	0.312
	Interaction (MxL)	2	3.273	0.11
CNP	Mycorrhizal_Type (M)	1	9.165	**0.022***
	Tree_Diversity (L)	2	0.839	0.538
	Interaction (MxL)	2	3.341	0.11

*All significant adjusted p values are highlighted in bold followed by significance level codes. *: $p \leq 0.05$. **: $p \leq 0.01$.*

Bala Singavarapu, Jianqing Du, Rémy Beugnon, Simone Cesarz, Nico Eisenhauer, Kai Xue, Yanfen Wang, Helge Bruelheide & Tesfaye Wubet

Table S6

Post-hoc analysis for effects of tree mycorrhizal type at each tree diversity level on the nutrient cycling functional compositional differences of soil co-occurring fungal and bacterial (whole) communities based on PERMANOVA with 999 permutations

Nutrient Cycle	Tree Diversity	df	F	R^2	pval.adj
C	Mono	1	3.407	0.091	**0.023***
	Two	1	3.657	0.097	**0.034***
	Multi	1	1.876	0.052	0.155
N	Mono	1	18.422	0.351	**0.004****
	Two	1	4.293	0.112	0.062
	Multi	1	1.778	0.05	0.222
P	Mono	1	13.226	0.28	**0.004****
	Two	1	5.012	0.128	**0.023***
	Multi	1	1.31	0.037	0.271
CN	Mono	1	11.049	0.245	**0.004****
	Two	1	4.324	0.113	**0.032***
	Multi	1	1.527	0.043	0.222
CP	Mono	1	13.771	0.288	**0.004****
	Two	1	6.981	0.17	**0.015***
	Multi	1	1.036	0.03	0.337
NP	Mono	1	16.469	0.326	**0.004****
	Two	1	4.994	0.128	**0.034***
	Multi	1	1.511	0.043	0.229
CNP	Mono	1	15.159	0.308	**0.004****
	Two	1	6.041	0.151	**0.018***
	Multi	1	1.286	0.036	0.271

*All significant adjusted p values are highlighted in bold followed by significance level codes. *: $p \leq 0.05$. **: $p \leq 0.01$.*

Table S7
Effects of tree mycorrhizal type and tree diversity level on the nutrient cycling functional compositional differences of the significantly soil-responsive modules of soil microbial networks based on PERMANOVA with 999 permutations

Nutrient Cycle	Factor	df	F	R^2	pval.adj
C	Mycorrhizal_Type (M)	1	3.852	0.034	**0.034***
	Tree_Diversity (L)	2	1.439	0.025	0.278
	Interaction (MxL)	2	2.252	0.04	0.061
N	Mycorrhizal_Type (M)	1	10.309	0.085	**0.014***
	Tree_Diversity (L)	2	1.439	0.024	0.288
	Interaction (MxL)	2	3.041	0.05	0.064
P	Mycorrhizal_Type (M)	1	6.692	0.058	**0.024***
	Tree_Diversity (L)	2	1.006	0.017	0.4
	Interaction (MxL)	2	2.831	0.049	0.06
CN	Mycorrhizal_Type (M)	1	7.384	0.062	**0.014***
	Tree_Diversity (L)	2	1.398	0.024	0.278
	Interaction (MxL)	2	2.993	0.051	**0.034***
CP	Mycorrhizal_Type (M)	1	5.58	0.047	**0.032***
	Tree_Diversity (L)	2	1.047	0.017	0.375
	Interaction (MxL)	2	4.993	0.083	**0.024***
NP	Mycorrhizal_Type (M)	1	8.103	0.068	**0.014***
	Tree_Diversity (L)	2	0.851	0.014	0.463
	Interaction (MxL)	2	3.614	0.061	**0.034***
CNP	Mycorrhizal_Type (M)	1	6.86	0.057	**0.027***
	Tree_Diversity (L)	2	1.087	0.018	0.375
	Interaction (MxL)	2	4.482	0.075	**0.024***

*All significant adjusted p values are highlighted in bold followed by significance level codes. *: $p \leq 0.05$. **: $p \leq 0.01$.*

Table S8
Post-hoc analysis for effects of tree mycorrhizal type at each tree diversity level on the nutrient cycling functional compositional differences of the significantly soil-responsive modules of soil microbial networks based on PERMANOVA with 999 permutations

Nutrient Cycle	Tree Diversity	df	F	R^2	pval.adj
C	Mono	1	2.393	0.066	0.074
	Two	1	3.489	0.093	**0.04***
	Multi	1	2.474	0.068	0.099
N	Mono	1	11.979	0.261	**0.008****
	Two	1	5.161	0.132	**0.032***
	Multi	1	2.049	0.057	0.134
P	Mono	1	6.712	0.165	**0.014***
	Two	1	4.735	0.122	**0.031***
	Multi	1	1.623	0.046	0.186
CN	Mono	1	7.380	0.178	**0.007****
	Two	1	4.727	0.122	**0.03***
	Multi	1	2.152	0.06	0.112
CP	Mono	1	6.516	0.161	**0.008****
	Two	1	6.846	0.168	**0.018***
	Multi	1	2.177	0.06	0.134
NP	Mono	1	9.481	0.218	**0.007****
	Two	1	5.275	0.134	**0.031***
	Multi	1	2.202	0.061	0.121
CNP	Mono	1	8.098	0.192	**0.007****
	Two	1	6.144	0.153	**0.026***
	Multi	1	2.321	0.064	0.112

*All significant adjusted p values are highlighted in bold followed by significance level codes. *: $p \leq 0.05$. **: $p \leq 0.01$.*

The functional potential of soil microbial communities and their sub-communities varies with tree mycorrhizal type and tree diversity

Bala Singavarapu, Jianqing Du, Rémy Beugnon, Simone Cesarz, Nico Eisenhauer, Kai Xue, Yanfen Wang, Helge Bruelheide & Tesfaye Wubet

Table S9

***envfit* analysis showing the significant gene families/Enzymes correlated to the ordination of significant modules of soil microbial networks**

Axis.1	Axis.2	Symbol	Name	Nutrient Cycle	Role	R^2	Tree diversity
0.324	-0.935	PQQ	Quinoprotein glucose dehydrogenase (PQQ. quinone)	Phosphorus	Inorganic P-solubilization and organic P-mineralization	0.978	Mono
0.333	-0.931	nirB	Nitrite reductase (NADH)	Nitrogen	DNRA (Denitrification and Dissimilatory Nitrate Reduction to Ammonia)	0.977	Mono
0.329	-0.916	nirK	Nitrite reductase (NO-forming)	Nitrogen	Denitrification / AnAmmOx	0.947	Mono
0.339	-0.905	nirA	Ferredoxin--nitrite reductase	Nitrogen	Assimilatory nitrate reduction	0.934	Mono
0.148	-0.988	HAO	Hydroxylamine reductase	Nitrogen	Nitrification / AnAmmOx	0.998	Mono
0.315	-0.947	PhoR	Histidine kinase	Phosphorus	P-starvation response regulation	0.996	Mono
0.689	0.718	tagl	Triacylglycerol lipase	Carbon	Glycerolipid metabolism	0.991	Mono
0.969	-0.195	phoA	Alkaline phosphatase	Phosphorus	Inorganic P-solubilization and organic P-mineralization	0.977	Mono
0.716	0.696	PHO	Acid phosphatase	Phosphorus	Inorganic P-solubilization and organic P-mineralization	0.998	Mono
0.767	-0.631	IMPA	Inositol-phosphate phosphatase	Phosphorus	Inorganic P-solubilization and organic P-mineralization	0.986	Mono
0.342	-0.898	appA	4-phytase	Phosphorus	Inorganic P-solubilization and organic P-mineralization	0.924	Mono
0.834	-0.544	glpQ	Glycerophosphodiester phosphodiesterase	Phosphorus	Inorganic P-solubilization and organic P-mineralization	0.991	Mono
0.332	-0.915	phnP	Phosphoribosyl 1.2-cyclic phosphate phosphodiesterase	Phosphorus	Inorganic P-solubilization and organic P-mineralization	0.947	Mono
0.936	0.335	AMY	Alpha-amylase	Carbon	Carbohydrate hydrolysis	0.989	Mono
0.724	0.688	ChiC	Chitinase	Nitrogen	Nitrogen metabolism	0.997	Mono
0.897	0.432	bglX	Beta-glucosidase	Carbon	cellulose hydrolysis	0.992	Mono
0.416	-0.907	pepA	Leucyl aminopeptidase	Nitrogen	Protein degradation	0.995	Mono
0.281	-0.949	pepN	Membrane alanyl aminopeptidase	Nitrogen	Glutathione metabolism	0.980	Mono
0.906	0.361	amiE	Amidase	Nitrogen	Degradation of aromatic and Nitrogen containing compounds	0.951	Mono

0.794	-0.571	URE	Urease	Nitrogen	Urea cycle	0.957	Mono
0.754	-0.649	ppa	Inorganic diphosphatase	Phosphorus	Inorganic P-solubilization and organic P-mineralization	0.989	Mono
0.717	-0.690	ppx	Exopolyphosphatase	Phosphorus	Inorganic P-solubilization and organic P-mineralization	0.991	Mono
0.279	-0.921	phnM	Alpha-D-ribose 1-methylphosphonate 5-triphosphate diphosphatase	Phosphorus	Inorganic P-solubilization and organic P-mineralization	0.927	Mono
0.628	0.765	lccA	Laccase	Carbon	Phenols and similar aromatic compounds Oxidation	0.980	Mono
0.728	0.631	PO	Peroxidase	Carbon	peroxidation of phenolic and non-phenolic substrates	0.928	Mono
-0.395	-0.890	PQQ	Quinoprotein glucose dehydrogenase (PQQ. quinone)	Phosphorus	Inorganic P-solubilization and organic P-mineralization	0.948	Two
-0.379	-0.919	nirB	Nitrite reductase (NADH)	Nitrogen	DNRA (Denitrification and Dissimilatory Nitrate Reduction to Ammonia)	0.988	Two
-0.252	-0.907	nirA	Ferredoxin--nitrite reductase	Nitrogen	Assimilatory nitrate reduction	0.887	Two
-0.409	-0.906	PhoR	Histidine kinase	Phosphorus	P-starvation response regulation	0.988	Two
-0.236	-0.898	phnN	Ribose 1.5-bisphosphate phosphokinase	Phosphorus	Inorganic P-solubilization and organic P-mineralization	0.862	Two
-0.327	-0.865	PhnI	Alpha-D-ribose 1-methylphosphonate 5-triphosphate synthase	Phosphorus	Inorganic P-solubilization and organic P-mineralization	0.855	Two
-0.567	0.821	tagl	Triacylglycerol lipase	Carbon	Glycerolipid metabolism	0.997	Two
-0.900	0.435	phoA	Alkaline phosphatase	Phosphorus	Inorganic P-solubilization and organic P-mineralization	0.999	Two
-0.576	0.815	PHO	Acid phosphatase	Phosphorus	Inorganic P-solubilization and organic P-mineralization	0.996	Two
-0.944	-0.326	IMPA	Inositol-phosphate phosphatase	Phosphorus	Inorganic P-solubilization and organic P-mineralization	0.997	Two
-0.468	-0.869	appA	4-phytase	Phosphorus	Inorganic P-solubilization and organic P-mineralization	0.974	Two
-0.433	0.893	phy	3-phytase	Phosphorus	Inorganic P-solubilization and organic P-mineralization	0.984	Two

-0.995	-0.083	glpQ	Glycerophosphodiester phosphodiesterase	Phosphorus	Inorganic P-solubilization and organic P-mineralization	0.998	Two
-0.339	-0.924	phnP	Phosphoribosyl 1.2-cyclic phosphate phosphodiesterase	Phosphorus	Inorganic P-solubilization and organic P-mineralization	0.968	Two
-0.297	-0.895	phnX	Phosphonoacetaldehyde hydrolase	Phosphorus	Inorganic P-solubilization and organic P-mineralization	0.888	Two
-0.868	0.492	AMY	Alpha-amylase	Carbon	Carbohydrate hydrolysis	0.995	Two
-0.647	0.759	ChiC	Chitinase	Nitrogen	Nitrogen metabolism	0.995	Two
-0.845	0.531	bglX	Beta-glucosidase	Carbon	cellulose hydrolysis	0.996	Two
-0.795	-0.572	CELB	Cellulase	Carbon	cellulose degradation	0.958	Two
-0.455	0.860	NAGLU	Alpha-N-acetylglucosaminidase	Carbon	Glucosamines degradation	0.947	Two
-0.341	-0.889	xynA	Endo-1.4-beta-xylanase	Carbon	Xylan degradation	0.907	Two
-0.322	-0.927	CBH1	Cellulose 1.4-beta-cellobiosidase (non-reducing end)	Carbon	cellulose degradation	0.962	Two
-0.573	-0.816	pepA	Leucyl aminopeptidase	Nitrogen	Protein degradation	0.993	Two
-0.390	-0.908	pepN	Membrane alanyl aminopeptidase	Nitrogen	Glutathione metabolism	0.976	Two
-0.652	0.753	amiE	Amidase	Nitrogen	Degradation of aromatic and Nitrogen containing compounds	0.993	Two
-0.926	-0.340	URE	Urease	Nitrogen	Urea cycle	0.974	Two
-0.952	-0.296	ppa	Inorganic diphosphatase	Phosphorus	Inorganic P-solubilization and organic P-mineralization	0.993	Two
-0.968	-0.246	ppx	Exopolyphosphatase	Phosphorus	Inorganic P-solubilization and organic P-mineralization	0.998	Two
-0.319	-0.888	phnM	Alpha-D-ribose 1-methylphosphonate 5-triphosphate diphosphatase	Phosphorus	Inorganic P-solubilization and organic P-mineralization	0.890	Two
-0.327	-0.865	phnJ	Alpha-D-ribose 1-methylphosphonate 5-phosphate C-P-lyase	Phosphorus	Inorganic P-solubilization and organic P-mineralization	0.855	Two
-0.544	0.834	lccA	Laccase	Carbon	Phenols and similar aromatic compounds Oxidation	0.992	Two
-0.818	0.561	PO	Peroxidase	Carbon	peroxidation of phenolic and non-phenolic substrates	0.984	Two
-0.317	0.944	PhoR	Histidine kinase	Phosphorus	P-starvation response regulation	0.991	Multi

-0.435	0.877	phnN	Ribose 1.5-bisphosphate phosphokinase	Phosphorus	Inorganic P-solubilization and organic P-mineralization	0.958	Multi
0.991	-0.117	tagl	Triacylglycerol lipase	Carbon	Glycerolipid metabolism	0.996	Multi
0.596	0.801	phoA	Alkaline phosphatase	Phosphorus	Inorganic P-solubilization and organic P-mineralization	0.997	Multi
0.995	-0.079	PHO	Acid phosphatase	Phosphorus	Inorganic P-solubilization and organic P-mineralization	0.997	Multi
-0.478	0.871	phnP	Phosphoribosyl 1.2-cyclic phosphate phosphodiesterase	Phosphorus	Inorganic P-solubilization and organic P-mineralization	0.988	Multi
0.961	0.213	AMY	Alpha-amylase	Carbon	Carbohydrate hydrolysis	0.970	Multi
0.977	-0.195	ChiC	Chitinase	Nitrogen	Nitrogen metabolism	0.993	Multi
0.978	0.198	bglX	Beta-glucosidase	Carbon	cellulose hydrolysis	0.996	Multi
0.946	0.190	NAGLU	Alpha-N-acetylglucosaminidase	Carbon	Glucosamines degradation	0.931	Multi
-0.306	0.937	pepN	Membrane alanyl aminopeptidase	Nitrogen	Glutathione metabolism	0.971	Multi
0.815	0.530	amiE	Amidase	Nitrogen	Degradation of aromatic and Nitrogen containing compounds	0.944	Multi
0.174	0.965	ppa	Inorganic diphosphatase	Phosphorus	Inorganic P-solubilization and organic P-mineralization	0.961	Multi
0.973	-0.194	lccA	Laccase	Carbon	Phenols and similar aromatic compounds Oxidation	0.985	Multi
0.971	0.165	PO	Peroxidase	Carbon	peroxidation of phenolic and non-phenolic substrates	0.970	Multi

Supplementary Material

The functional potential of soil microbial communities and their sub-communities varies with tree mycorrhizal type and tree diversity

Bala Singavarapu, Jianqing Du, Rémy Beugnon, Simone Cesarz, Nico Eisenhauer, Kai Xue, Yanfen Wang, Helge Bruelheide & Tesfaye Wubet

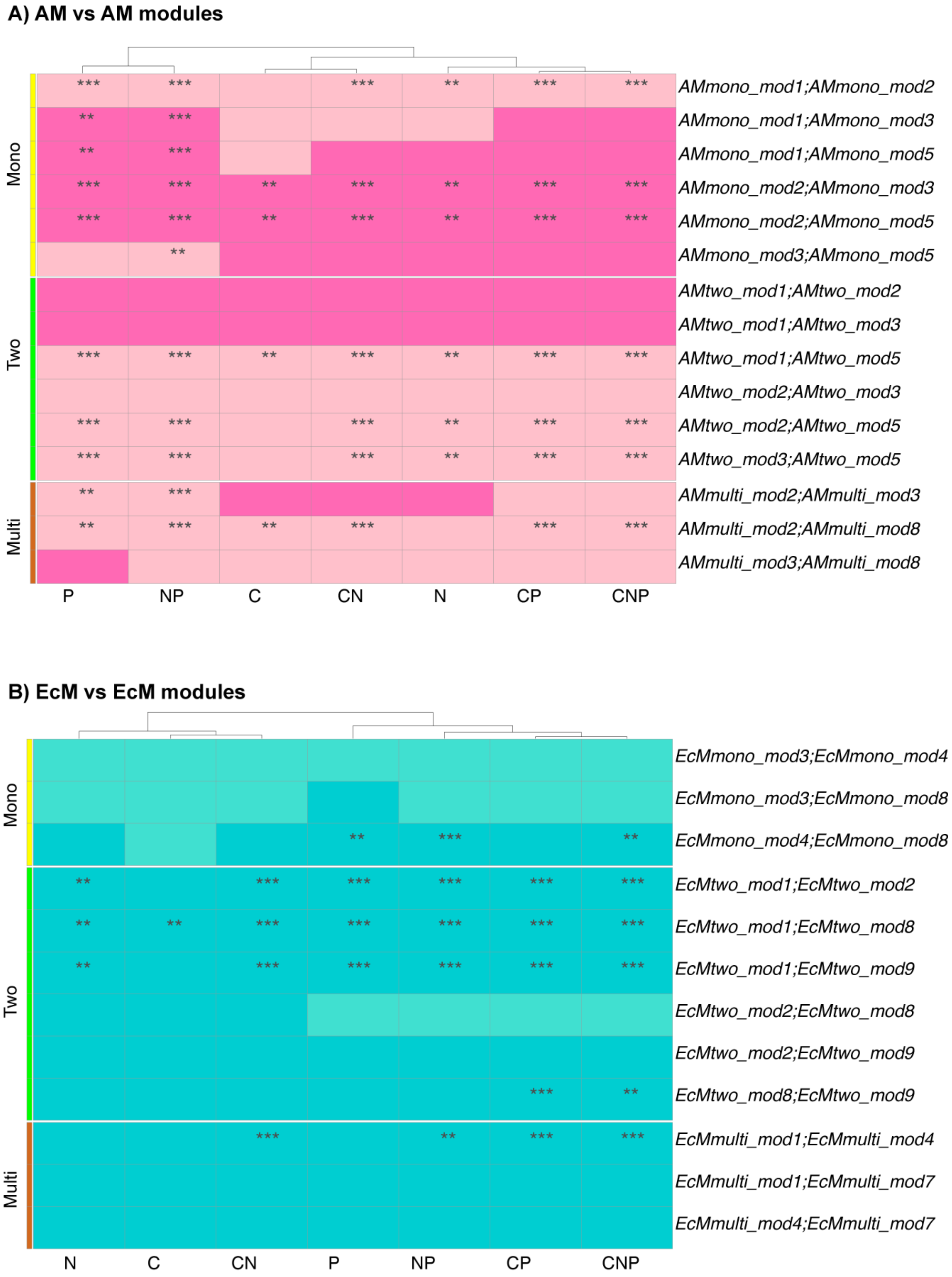

Fig. S1: Heat map of pairwise comparisons of significantly soil-responding modules within the tree mycorrhizal type along the tree diversity levels. (A) Modules of AM tree mycorrhizal type. (B) EcM tree mycorrhizal type. The asterisks show the p-value significance level, $*p \leq 0.05$, $**p \leq 0.01$, $***p \leq 0.001$, $****p \leq 0.0001$.
